# The geometry of *G* × *E*: How scaling and endogenous treatment effects shape interaction direction

**DOI:** 10.1371/journal.pgen.1012073

**Published:** 2026-04-01

**Authors:** Michal Sadowski, Andy W. Dahl, Noah Zaitlen, Richard Border

**Affiliations:** 1 Bioinformatics Interdepartmental Program, University of California Los Angeles, Los Angeles, California, United States of America; 2 Section of Genetic Medicine, Department of Medicine, University of Chicago, Chicago, Illinois, United States of America; 3 Department of Neurology, University of California Los Angeles, Los Angeles, California, United States of America; 4 Department of Computational Medicine, David Geffen School of Medicine, University of California Los Angeles, Los Angeles, California, United States of America; 5 Department of Human Genetics, David Geffen School of Medicine, University of California Los Angeles, Los Angeles, California, United States of America; 6 Department of Computational Biology, Carnegie Mellon University, Pittsburgh, Pennsylvania, United States of America; Yale University, UNITED STATES OF AMERICA

## Abstract

Gene-environment interaction (*G* × *E*) studies hold promise for identifying genetic loci mediating the effects of environmental risk on disease. However, interpretation of *G* × *E* effects is often confounded by two fundamental issues: the dependence of interaction estimates on outcome scale and the presence of endogenous treatment effects, in which genetic liability influences environmental exposure. These factors can induce apparent *G* × *E* signals—even when genetic and environmental contributions are purely additive on an unobserved scale. In this work, we demonstrate that any monotone convex transformation of an outcome induces *sign-consistent G × E* effects: the sign of the interaction term aligns with the sign of the corresponding main genetic effect. Convex transformations are a broad class of functions that include many commonly used data transformations, such as exponential and logarithmic functions, the square root, and other power transformations. We further show that endogenous treatment effects, modeled as threshold-based interventions, generate *G* × *E* effects with a similar directional signature. Exploiting this property, we propose a simple diagnostic: sign consistency across *G* × *E* estimates can signal when interactions are driven by outcome scaling or exposure endogeneity. We validate our framework in the UK Biobank using transcriptome-wide interaction studies (TxEWAS) across multiple trait–environment pairs, observing widespread sign consistency in some settings—suggesting confounding by scaling or treatment bias. Our results provide both a theoretical foundation and a practical tool for interpreting *G* × *E* findings, enabling researchers to assess whether the observed *G* × *E* signal may depend substantially on outcome scaling or be influenced by exposure endogeneity.

## Author summary

Gene-environment interaction (*G* × *E*) studies examine the extent to which genetic differences modulate environmental impacts on individuals’ health outcomes. However, their results depend on how these outcomes are measured or modeled, and are often confounded by endogenous treatment effects, where exposure to an environment depends on the health outcome itself (for example, individuals with high blood pressure are more likely to receive blood pressure reducing medications). We demonstrate that both a wide class of scaling functions and endogenous treatment effects induce *sign-consistent G × E*: the direction of the interaction aligns with the direction of the main genetic effect. This property can be used as a diagnostic to assess when an apparent *G* × *E* signal could be driven by outcome scaling or exposure endogeneity.

## Introduction

Individuals exhibit substantial phenotypic heterogeneity in response to environmental perturbations. Part of this heterogeneity arises from individual differences in genetic background and is referred to as gene-environment interaction (*G* × *E*). Several interactions identified to date have important implications for human health. For example: (1) dietary treatment prevents symptoms of phenylketonuria—a genetic disorder caused by mutations in the *PAH* gene [1]; (2) physical activity blunts the effects of obesity risk variants in the fat mass and obesity-associated gene, *FTO* [2]; (3) a variant of the *NAT2* gene elevates the risk of bladder cancer in smokers [3]; and (4) a multitude of gene polymorphisms have been shown to impact drug response or toxicity [4–8]. These examples showcase the potential of *G* × *E* discovery to enhance disease prevention and management, to enable design of individualized treatments that are safer and more effective, and, more generally, to advance our understanding of disease etiology. To unlock this potential, many methods for *G* × *E* detection have been developed [9–12] and they are continually being optimized. Most recent approaches enable genome-wide *G* × *E* screens in large-scale studies of human populations [13–15].

**Fig 1:**
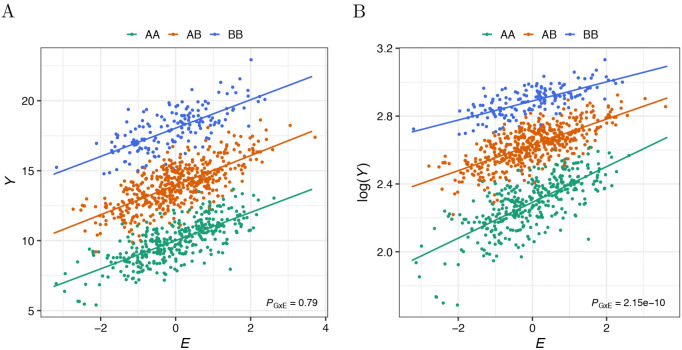
*G × E* effect induced by log-transformation of outcome *Y.* A: Depiction of the effects of the genetic variant *G* (with reference allele A and alternative allele B, MAF = 0.4), the environmental factor *E* (drawn from a standard normal distribution), and the interaction between the two (*G* × *E*) on the outcome *Y* (generated as Y~𝒩(10+4G+E,1) for 1,000 samples). The p-value for the *G* × *E* effect (*P*_GxE_) is given in the right lower corner. B: Depiction of the same effects on the log-transformed *Y*. Whereas the *G* × *E* effect is not detectable for *Y* (A), it is detectable for the log-transformed *Y* (B).

Standard statistical methods assess *G* × *E* by comparing additive and non-additive models of genetic and environmental effects. Although valuable for exploratory analysis, this definition depends on modeling assumptions and might not reflect biological mechanisms of interaction [16,17]. Here, we focus on two fundamental issues that complicate the interpretation of current *G* × *E* approaches: (1) dependence on phenotype scale and (2) endogenous treatment effects. In case (1), the detection of an interaction effect and its direction depend on the scale on which the outcome is measured, or to which it might be transformed [18–21]. For example, even though a genetic variant *G* and an environmental factor *E* impact an outcome *Y* additively, an interaction test performed on *Y* that has been log-transformed (e.g., as part of quality control processing) can yield a highly significant *G* × *E* effect (Fig 1). More generally, many interaction effects can be induced or removed by monotonic non-linear transformations of the data. In case (2), exposure and genetic liability are causally intertwined. For example, imagine that a treatment is administered to taper the level of a heritable phenotype when it crosses some threshold (e.g., statins may be prescribed to lower low-density lipoprotein (LDL) cholesterol levels). In this case, exposure to the intervention is related to genetic factors influencing phenotype. Such endogenous treatment effects can result in apparent *G* × *E*, even when gene and environment act additively on the observed scale. As a result, most *G* × *E* findings require the caveat that they are specific to a particular measurement or may be a consequence of endogenous treatment effects [22–25].

Not all observed interactions require these caveats. For instance, there is an extreme form of interaction, also known as a *crossover* effect, in which the direction of an association (rather than only its magnitude) depends on a moderating factor. This type of interaction cannot be eliminated by a monotonic transformation [16] and, when sufficiently large, has a relatively straightforward interpretation [17]. Other statistical interactions, however, can in principle be removed by a monotonic transformation—meaning that, after transforming the outcome, the data are adequately described by an additive model.

Here, we demonstrate that monotone convex transformations of an outcome induce *sign-consistent G × E*, where the direction of the interaction effects is determined by the sign of the corresponding main effects. We further show that endogenous treatment effects, modeled as threshold-based interventions, also generate sign-consistent *G* × *E*. Finally, we discuss examples of non-convex transformations, like the logistic function, showing why and under what circumstances they induce this particular type of interaction effects. Our results indicate that a simple examination of sign consistency across detected *G* × *E* can rule out the possibility that all interactions have been induced by a monotone convex scaling of the outcome or endogenous treatment effects. Another consequence of this result is that if *G* × *E* signal is not sign-consistent, it cannot be eliminated by a monotone convex transformation.

Monotone convex transformations describe a broad class of functions that include many of the most commonly used data transformations in genetic studies and data analysis in general. For example, Box–Cox transformations, which are widely used to reduce departures from normality, are convex. Examples of transformations that are convex down include the square function, power transformations with even exponents, and the exponential function. Transformations that are convex up include the logarithm, the square root, and power transformations with exponents between zero and one. Many more commonly used transformations are locally convex; for instance, the logistic function and the hyperbolic tangent are convex down on one half of their domain and convex up on the other. We restrict our attention to monotone transformations as they preserve (or completely reverse) the order of data values.

We demonstrate the usefulness of sign consistency examination in real data, as our analysis of this property identifies that statin use induces false positive gene-age interaction effects on LDL cholesterol levels.

## Results

### Sign-consistent interaction property

We investigate the relationship between the signs of regression coefficients estimated in the *G* × *E* model across multiple genetic variants. More concretely, consider testing two haploid variants, *G*_1_ and *G*_2_, for an interaction with a binary environmental exposure *E* against phenotype *Y* in two single-variant regressions:


$$\begin{array}{cc} & Y={\hat{\mu }}_{1}+{\hat{\alpha }}_{1}E+{\hat{\beta }}_{1}G_{1}+{\hat{\gamma }}_{1}G_{1}E+\varepsilon_{1},\\ & Y={\hat{\mu }}_{2}+{\hat{\alpha }}_{2}E+{\hat{\beta }}_{2}G_{2}+{\hat{\gamma }}_{2}G_{2}E+\varepsilon_{2}, \end{array}$$

where ${\hat{\mu }}_{i}$, ${\hat{\alpha }}_{i}$, ${\hat{\beta }}_{i}$ and ${\hat{\gamma }}_{i}$ are coefficients estimated in regression *i*, and $\varepsilon_{i}$ represents homoskedastic residual variation. Critically, neither of these models needs to accurately describe causal relationships; we just assume we can fit these regression models and have well-behaved errors.

Next, consider the same two tests performed for the same phenotype *Y* measured on a different scale:


$$\begin{array}{cc} & \varphi (Y)={\hat{\mu }}_{1}^{\varphi }+{\hat{\alpha }}_{1}^{\varphi }E+{\hat{\beta }}_{1}^{\varphi }G_{1}+{\hat{\gamma }}_{1}^{\varphi }G_{1}E+\varepsilon_{1}^{\varphi },\\ & \varphi (Y)={\hat{\mu }}_{2}^{\varphi }+{\hat{\alpha }}_{2}^{\varphi }E+{\hat{\beta }}_{2}^{\varphi }G_{2}+{\hat{\gamma }}_{2}^{\varphi }G_{2}E+\varepsilon_{2}^{\varphi }, \end{array}$$

where φ is the map from the scale of the former measurement of *Y* to the scale of the latter measurement of *Y*, and the corresponding coefficients and residuals are marked with superscript φ.

We demonstrate that the signs of interaction effects, ${\hat{\gamma }}_{1}^{\varphi }$ and ${\hat{\gamma }}_{2}^{\varphi }$, induced by a monotone convex transformation φ depend on the signs of the main genetic effects, ${\hat{\beta }}_{1}^{\varphi }$ and ${\hat{\beta }}_{2}^{\varphi }$. The interaction effects satisfy a precise sign rule:

**Theorem 1** (Sign-consistent interaction property). *Assume measurement Y has homogeneous variance and exhibits no G × E effects (${\hat{\gamma }}_{1}={\hat{\gamma }}_{2}=0$) on the original scale, with G and E independent, mean-centered, and having finite variance. If* φ *is a monotone convex transformation, then the G × E effects ${\hat{\gamma }}_{1}^{\varphi }$ and ${\hat{\gamma }}_{2}^{\varphi }$ satisfy:*


$$sgn({\hat{\gamma }}_{i}^{\varphi })=sgn(\varphi^{\prime \prime })\cdot sgn({\hat{\alpha }}_{i}^{\varphi })\cdot sgn({\hat{\beta }}_{i}^{\varphi }),$$ (1)

*where* $sgn(\varphi^{\prime \prime })$ *denotes the sign of the second derivative of φ (positive for convex down, negative for convex up).*

Note that if we assume that the alleles of *G*_1_ and *G*_2_ are encoded so that their main effects have the same direction (i.e., $sgn({\hat{\beta }}_{1}^{\varphi })=sgn({\hat{\beta }}_{2}^{\varphi })$), the above property becomes:


$$sgn({\hat{\gamma }}_{1}^{\varphi })=sgn({\hat{\gamma }}_{2}^{\varphi }),$$ (2)

which we call *sign consistency*. By contraposition, if the homoskedasticity and ${\hat{\gamma }}_{1}={\hat{\gamma }}_{2}=0$ assumptions hold but property (2) does not, non-zero *G* × *E* effects ${\hat{\gamma }}_{1}^{\varphi }$ and ${\hat{\gamma }}_{2}^{\varphi }$ are not both induced by a monotone convex scaling of *Y*.

Though we focus on the simple case of haploid genotypes and binary environmental exposures in the primary text, these results apply to diploid genotypes and continuous environmental exposures.

**Corollary 1** (Sign consistency for diploid genotypes). *The sign rule (1) extends to diploid genotypes G∈{0,1,2} under Hardy-Weinberg equilibrium, with E any random variable satisfying the independence and moment conditions above.*

We further generalize this result to allow for moderate *G*-*E* correlations:

**Theorem 2** (Sign consistency for correlated *G* and *E*). *When G and E are correlated, the induced interaction effect includes an additional correlation term:*


$${\hat{\gamma }}^{\varphi }\approx \gamma_{\text{corr}}+\alpha \beta \cdot 𝔼[\varphi^{\prime \prime }(\varepsilon )],$$

*where* $\gamma_{\text{corr}}$ *arises from the non-orthogonality of predictors. The sign rule (1) holds when the transformation effect dominates:* $\left|\alpha \beta \cdot 𝔼[\varphi^{\prime \prime }]|>|\gamma_{\text{corr}}\right|$.

**Corollary 2** (Dominance of transformation effect). *For small gene-environment correlations or strong curvature $\left|\varphi^{\prime \prime }\right|$, the sign rule approximately holds. In practice, if observed interactions are strongly sign-consistent across many variants, the transformation effect likely dominates any correlation-induced deviations.*

Finally, we show that endogenous treatment effects—where treatment is assigned based on a phenotype threshold—also induce sign-consistent interactions:

**Theorem 3** (Opposite-sign rule for endogenous treatment). *Consider an environmental exposure E assigned when phenotype Y exceeds a threshold t, with treatment effect* α *on Y. For a genetic variant with effect $\beta_{j}$ on Y, if the treatment threshold exceeds the reference mean ($P_{0}=t-\mu >0$), the induced interaction satisfies:*


$$sgn({\hat{\gamma }}_{j})=-sgn({\hat{\beta }}_{j}).$$

We prove these results rigorously in S1 Appendix.

Generally, in abstraction from this two-variant example, we demonstrate that if there is a scale, on which phenotype *Y* has homogeneous variance across values of environmental factor *E* and genetic variant *G*, and those factors have only additive effects on *Y*, then the direction of the *G* × *E* effect estimated for a measurement that is a monotone convex transformation of *Y* depends on the direction of the corresponding main genetic effect.

We, therefore, propose to examine observed *G* × *E* effects for the sign consistency property, as it provides a means to exclude the family of monotone convex transformations as the sole source of these effects.

### Sketch of argument

Suppose that phenotype *Y* has homogeneous variance across values of environmental factor *E* and genotype *G*:


$$\forall_{a,b}Y{|}_{E=a,G=b}\sim 𝒟(\nu_{a,b},\sigma^{2}),$$ (3)

where 𝒟 is a symmetric distribution with mean *ν* and variance $\sigma^{2}$. We fit the following linear regression model to test the effect of an interaction between *G* and *E* on this phenotype:


$$Y=\hat{\mu }+\hat{\alpha }E+\hat{\beta }G+\hat{\gamma }GE+\varepsilon ,$$ (4)

where $\hat{\mu }$, $\hat{\alpha }$, $\hat{\beta }$ and $\hat{\gamma }$ are estimated coefficients and *ε* is the error. We consider a simplified example, where *E* and *G* are binary variables, which corresponds to the case of a haploid genetic variant and a binary environmental exposure. Importantly, our reasoning is not contingent on whether this model is correct, only that the homoskedasticity condition (3) holds. The coefficients in (4) can be related to the empirical conditional expectations:


$$\begin{array}{cc} & \hat{\mu }=P_{0,0},\\ & \hat{\alpha }=P_{1,0}-P_{0,0},\\ & \hat{\beta }=P_{0,1}-P_{0,0},\\ & \hat{\gamma }=(P_{1,1}-P_{1,0})-(P_{0,1}-P_{0,0}), \end{array}$$

where $\begin{array}{c} P_{a,b}:=\hat{𝔼}[Y|E=a,G=b] \end{array}$. For example, *P*_0,1_ is the average value of phenotype *Y* in individuals with genotype *G* = 1 who are not exposed to environmental factor *E* (i.e., *E* = 0).

**Fig 2:**
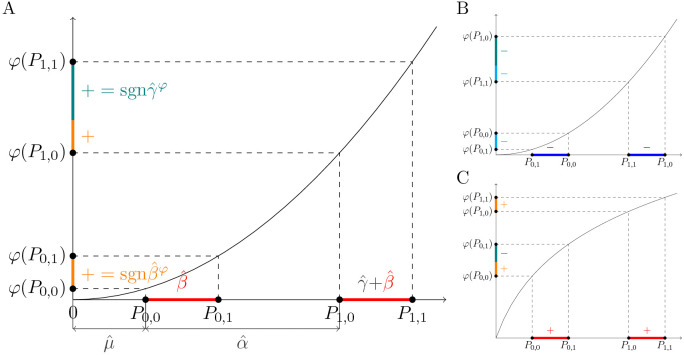
Monotone convex transformations of the outcome induce sign-consistent *G* × *E* effects in interaction tests. A: The x-axis shows the intercept ($\hat{\mu }$), the main effect of *E* ($\hat{\alpha }$), the main effect of *G* ($\hat{\beta }$) and the *G* × *E* effect ($\hat{\gamma }$) estimated by regressing *E*, *G* and *GE* on phenotype *Y*. If, as assumed here, $\hat{\alpha }$ and $\hat{\beta }$ are positive and $\hat{\gamma }$ is null, a similar regression on this phenotype transformed with an increasing convex down function φ will yield the main effect of *G* (${\hat{\beta }}^{\varphi }$) and the *G* × *E* effect (${\hat{\gamma }}^{\varphi }$) that are positive. The sign of the *G* × *E* effect can be calculated by $sgn({\hat{\gamma }}^{\varphi })=sgn(\varphi (P_{1,1})-\varphi (P_{1,0})-(\varphi (P_{0,1})-\varphi (P_{0,0})))$, where $\begin{array}{c} P_{a,b}:=\hat{𝔼}[Y|E=a,G=b] \end{array}$. B: Similar to A, but shows the signs of ${\hat{\beta }}^{\varphi }$ and ${\hat{\gamma }}^{\varphi }$ when $\hat{\beta }$ is negative. C: Similar to A, but shows the signs of ${\hat{\beta }}^{\varphi }$ and ${\hat{\gamma }}^{\varphi }$ when φ is increasing convex up. The colored segments on the x-axis indicate the sign of $\hat{\beta }$—red if positive and blue if negative. Orange and cyan segments on the y-axis denote, respectively, positive and negative values of $\varphi (P_{0,1})-\varphi (P_{0,0})$ or $\varphi (P_{1,1})-\varphi (P_{1,0})$, whichever has smaller magnitude. The green segment represents the difference between these two differences, which may be positive or negative depending on the transformation.

Without loss of generality, suppose further that coefficients $\hat{\mu }$, $\hat{\alpha }$ and $\hat{\beta }$ are estimated to be positive, and that the estimate of the *G* × *E* effect $\hat{\gamma }$ is zero, as depicted on the x-axis of Fig 2A. Consider now a regression similar to (4), but performed on phenotype *Y* measured on a different than the original scale:


$$\varphi (Y)={\hat{\mu }}^{\varphi }+{\hat{\alpha }}^{\varphi }E+{\hat{\beta }}^{\varphi }G+{\hat{\gamma }}^{\varphi }GE+\varepsilon^{\varphi },$$ (5)

where φ is the map from the original scale of *Y* to the new scale.

If we assume that φ is increasing convex down (Fig 2A), then the signs of ${\hat{\beta }}^{\varphi }$ and ${\hat{\gamma }}^{\varphi }$ can be related to points *P*_*a*,*b*_ as:


$$sgn({\hat{\beta }}^{\varphi })=sgn(\varphi (P_{0,1})-\varphi (P_{0,0})),$$ (6)

$$sgn({\hat{\gamma }}^{\varphi })=sgn(\varphi (P_{1,1})-\varphi (P_{1,0})-(\varphi (P_{0,1})-\varphi (P_{0,0}))).$$ (7)

That is, with the above assumptions, the direction of the effects estimated for the scaled phenotype φ(Y) can be expressed using quantities *P*_*a*,*b*_ defined on the original scale of *Y* (see Methods for a derivation of this fact). Looking at Fig 2A, it is easy to see that in our example:

1. The signs of differences $\varphi (P_{0,1})-\varphi (P_{0,0})$ and $\varphi (P_{1,1})-\varphi (P_{1,0})$ are the same, and, by (6), follow the sign of ${\hat{\beta }}^{\varphi }$.
2. The magnitude of $\varphi (P_{1,1})-\varphi (P_{1,0})$ is larger than the magnitude of $\varphi (P_{0,1})-\varphi (P_{0,0})$.

From (7) and these two facts it follows that the sign of ${\hat{\gamma }}^{\varphi }$ is positive. Note that this will be true for any genetic variant whose main effect, ${\hat{\beta }}^{\varphi }$, tested in (5) is positive. If, on the other hand, ${\hat{\beta }}^{\varphi }$ is negative, the *G* × *E* effect, ${\hat{\gamma }}^{\varphi }$, will be negative (Fig 2B). In general, we have the following relation:


$$sgn({\hat{\gamma }}^{\varphi })=sgn({\hat{\beta }}^{\varphi }).$$

Applying a similar argument, it can be shown that when φ is increasing convex up, the opposite relation, $sgn({\hat{\gamma }}^{\varphi })=-sgn({\hat{\beta }}^{\varphi })$, holds (Fig 2C). In general, the direction of this relation depends on whether φ is convex down or convex up, and on the sign of ${\hat{\alpha }}^{\varphi }$, that is, the estimated effect of environmental factor *E* (Table 1). A complete proof discussing these cases is given in Methods.

**Table 1:** Monotone convex transformations induce *G* × *E* effects (${\hat{\gamma }}^{\varphi }$) whose directions are consistent with the directions of the observed main effects (${\hat{\alpha }}^{\varphi }$, ${\hat{\beta }}^{\varphi }$). In general, $sgn({\hat{\gamma }}^{\varphi })=sgn(\varphi^{\prime \prime })\cdot sgn({\hat{\alpha }}^{\varphi })\cdot sgn({\hat{\beta }}^{\varphi })$. Note that functions like $\sqrt{x}$ and log(x) must be defined on appropriate domains (e.g., *x* > 0).

	Example functions	${\hat{\alpha }}^{\varphi }>0$	${\hat{\alpha }}^{\varphi }<0$
φ convex down ($\varphi^{\prime \prime }>0$)	$\begin{array}{c} e^{x},e^{-x},x^{2} \end{array}$	$sgn({\hat{\gamma }}^{\varphi })=sgn({\hat{\beta }}^{\varphi })$	$sgn({\hat{\gamma }}^{\varphi })=-sgn({\hat{\beta }}^{\varphi })$
φ convex up ($\varphi^{\prime \prime }<0$)	log(x), $\sqrt{x}$, $\begin{array}{c} -x^{2} \end{array}$	$sgn({\hat{\gamma }}^{\varphi })=-sgn({\hat{\beta }}^{\varphi })$	$sgn({\hat{\gamma }}^{\varphi })=sgn({\hat{\beta }}^{\varphi })$

### Endogenous treatment effects

Suppose that the environmental factor *E* tested in the *G* × *E* regression model is a treatment. Suppose that this treatment is administered to taper the level of a heritable phenotype when it crosses some threshold—e.g., the statin therapy for individuals with high LDL cholesterol. In this case, exposure to the intervention is related to genetic factors influencing phenotype—which we refer to as *endogenous treatment effects*. As shown in [8], endogenous treatment effects can cause false discoveries when the observed levels of the phenotype (subjected to treatment) are tested for *G* × *E*. Following, we prove that the *G* × *E* effects induced in a simple model of endogenous treatment effects are sign-consistent.

Consider the following model of phenotype *Y*:


$$Y=\sum_{i}\beta_{i}G_{i}+\varepsilon ,\;\varepsilon \sim 𝒩(0,\sigma_{\varepsilon }^{2}),$$

where, again, *G*_*i*_ indicates the presence of an alternative allele at haploid variant *i*, $\beta_{i}$ is the effect of this allele on *Y*, and *ε* is the environmental noise. We assume that the environmental noise is homoskedastic ($\forall_{i}Var[\varepsilon |G_{i}=0]=Var[\varepsilon |G_{i}=1]$).

Suppose that if the level of *Y* is high, an individual is administered treatment *E*:


$$\begin{array}{c} E=\left\{ \begin{array}{cc} 1 & \text{if }Y>t,\\ 0 & \text{otherwise}, \end{array}\right. \end{array}$$

where *t* is some threshold. When applied, treatment *E* changes the level of *Y* by $\begin{array}{c} \alpha \end{array}$:


$$\tilde{Y}=Y+\alpha E.$$ (8)

**Claim 1** (Endogenous treatment effect interaction sign property). *Suppose that we observe phenotype $\tilde{Y}$ and test the effect ${\hat{\gamma }}_{j}$ of the interaction between variant G*_*j*_ *and environmental factor E on this phenotype:*


$$\tilde{Y}={\hat{\mu }}_{j}+{\hat{\alpha }}_{j}E+{\hat{\beta }}_{j}G_{j}+{\hat{\gamma }}_{j}G_{j}E+\varepsilon_{j},$$ (9)

*Then the direction of the estimated G × E effect,* ${\hat{\gamma }}_{j}$*, is opposite to the direction of the main genetic effect,* ${\hat{\beta }}_{j}$.

As in *Sketch of argument*, we define quantities *P*_0_ and *P*_1_ on the scale of Y, which, transformed, can be used to compute the main genetic effect, ${\hat{\beta }}_{j}$, and the *G* × *E* effect, ${\hat{\gamma }}_{j}$, on the scale of $\tilde{Y}$. Note that in this case, not only the signs, but also the values of these effects can be expressed in terms of *P*_0_ and *P*_1_ transformed by a certain function. We investigate the properties of this function to determine the properties of ${\hat{\beta }}_{j}$ and ${\hat{\gamma }}_{j}$. Specifically, we define $P_{0}:=t-𝔼[Y|G_{j}=0]$ and $P_{1}:=t-𝔼[Y|G_{j}=1]$, and show (Methods) that the main genetic effect ${\hat{\beta }}_{j}$ and the *G* × *E* effect ${\hat{\gamma }}_{j}$ estimated in (9) can be related to these points as:


$$\begin{array}{cc} & 𝔼[{\hat{\beta }}_{j}]=\lambda (P_{0})-\lambda (P_{1})+P_{0}-P_{1},\\ & 𝔼[{\hat{\gamma }}_{j}]=\lambda (-P_{1})-\lambda (-P_{0})-(\lambda (P_{0})-\lambda (P_{1})), \end{array}$$

where $\begin{array}{c} \lambda \end{array}$ is the inverse Mill’s Ratio (λ(x):=ϕ(x)/Φ(x), where $\begin{array}{c} \varphi \end{array}$ and Φ are the standard normal probability density function and cumulative distribution function, respectively). Importantly, $\begin{array}{c} \lambda \end{array}$ is decreasing and strictly convex down [26].

**Fig 3:**
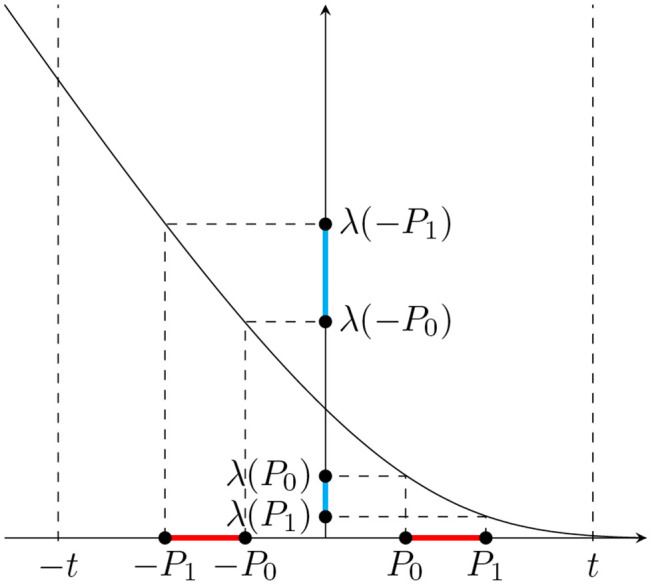
Endogenous treatment effects induce sign-consistent *G* × *E* effects. The x-axis shows the quantities $P_{0}:=t-𝔼[Y|G=0]$ and $P_{1}:=t-𝔼[Y|G=1]$ defined for phenotype *Y* affected by the haploid genetic variant *G*. The main effect of *G* and the effect of *GE* on phenotype *Y*, after treatment *E* is applied to reduce levels of *Y* that exceed threshold *t*, can be expressed as functions of *P*_0_ and *P*_1_ and their images under the inverse Mill’s Ratio $\lambda (P_{0})$ and $\lambda (P_{1})$. The signs of those functions are dependent.

Suppose that $0<P_{0}<P_{1}$. Then, looking at Fig 3, we see that:

1. The difference $\lambda (P_{0})-\lambda (P_{1})$ has the opposite sign to difference $P_{0}-P_{1}$.
2. The magnitude of $P_{0}-P_{1}$ is larger than the magnitude of $\lambda (P_{0})-\lambda (P_{1})$.
3. The difference $\lambda (-P_{1})-\lambda (-P_{0})$ has the same sign as difference $\lambda (P_{0})-\lambda (P_{1})$.
4. The magnitude of $\lambda (-P_{1})-\lambda (-P_{0})$ is greater than the magnitude of $\lambda (P_{0})-\lambda (P_{1})$.

From our assumption that $0<P_{0}<P_{1}$ and facts 1 and 2 above, it follows that the sign of ${\hat{\beta }}_{j}$ is negative. From the same assumption and facts 1, 3 and 4, it follows that the sign of ${\hat{\gamma }}_{j}$ is positive. Note that once the sign of difference $P_{0}-P_{1}$ is established, the signs of ${\hat{\beta }}_{j}$ and ${\hat{\gamma }}_{j}$ can be determined based on the properties of λ. In our example, $P_{0}-P_{1}$ is negative, which makes ${\hat{\beta }}_{j}$ negative and ${\hat{\gamma }}_{j}$ positive. On the other hand, when $P_{0}-P_{1}$ is positive, ${\hat{\beta }}_{j}$ is positive and ${\hat{\gamma }}_{j}$ is negative. Therefore, we have shown that *G* × *E* effects induced by endogenous treatment effects (modeled as in (8)) have opposite directions to the corresponding main genetic effects. That is, for any *j*, $sgn({\hat{\gamma }}_{j})=-sgn({\hat{\beta }}_{j})$.

The same property holds when treatment *E* is administered whenever the level of *Y* is below threshold *t* (Methods).

### Non-convex transformations

An arbitrary non-linear scaling of an outcome may or may not induce sign-consistent *G* × *E*. To provide more intuition on this, we discuss properties of *G* × *E* that can be produced by two examples of non-convex scaling commonly used in genetic analyses: (1) the logistic function and (2) the inverse normal transformation (INT).

Specifically, we are interested in the relationship between the signs of the main effect ${\hat{\beta }}^{\varphi }$ of genetic variant *G* and the effect ${\hat{\gamma }}^{\varphi }$ of the interaction between *G* and environmental factor *E* on φ-transformed phenotype *Y* in the linear regression:


$$\varphi (Y)={\hat{\mu }}^{\varphi }+{\hat{\alpha }}^{\varphi }E+{\hat{\beta }}^{\varphi }G+{\hat{\gamma }}^{\varphi }GE+\varepsilon^{\varphi }.$$ (10)

Following *Sketch of argument*, we assume that *E* and *G* are binary and that *Y* (on the original scale) has homogeneous variance and does not exhibit *G* × *E* effects, meaning that the linear regression:


$$Y=\hat{\mu }+\hat{\alpha }E+\hat{\beta }G+\hat{\gamma }GE+\varepsilon$$

yields $\hat{\gamma }=0$.

As demonstrated earlier, given these assumptions, ${\hat{\beta }}^{\varphi }$ and ${\hat{\gamma }}^{\varphi }$ can be defined in terms of the empirical conditional means of untransformed *Y*:


$${\hat{\beta }}^{\varphi }=\int [\varphi (P_{0,1}+\varepsilon )-\varphi (P_{0,0}+\varepsilon )]f(\varepsilon )d\varepsilon ,$$ (11)

$${\hat{\gamma }}^{\varphi }=\int [\underset{:=A(\varepsilon )}{\underbrace{\varphi (P_{1,1}+\varepsilon )-\varphi (P_{1,0}+\varepsilon )}}-(\underset{:=B(\varepsilon )}{\underbrace{\varphi (P_{0,1}+\varepsilon )-\varphi (P_{0,0}+\varepsilon )}})]f(\varepsilon )d\varepsilon =\int [A(\varepsilon )-B(\varepsilon )]f(\varepsilon )d\varepsilon ,$$ (12)

where $P_{a,b}:=\hat{𝔼}[Y|E=a,G=b]$ and *f* is the PDF of *ε*.

#### Case study: the logistic function.

Let φ be a logistic function:


$$\varphi (x)=\frac{1}{1+e^{-(x-x_{0})}}.$$ (13)

**Fig 4:**
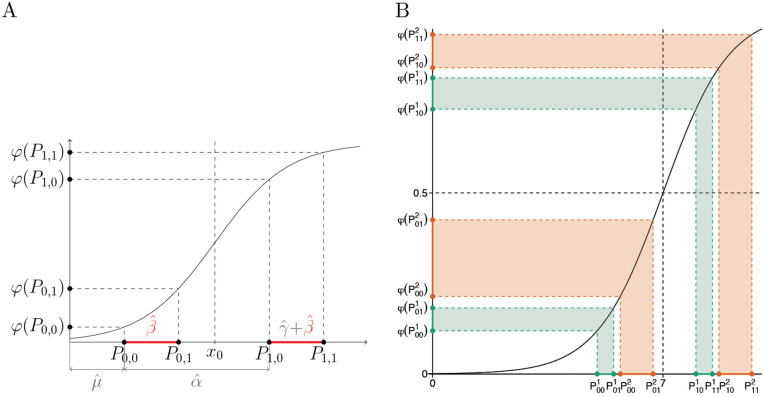
Logistic transformation of the outcome induces *G* × *E* effects that may or may not be sign-consistent. A: Depiction of the effect that the logistic transformation $\varphi (x)=1/(1+e^{-(x-x_{0})})$ of the outcome may have on the regression-based *G* × *E* test. Compare with Fig 2. B: An example of two genetic variants (green and orange) with positive effects on the phenotype that after transforming this phenotype with the logistic function $\varphi (x)=1/(1+e^{-(x-7)})$ exhibit *G* × *E* effects of opposite directions. Compare with Fig 2.

Using definitions (11) and (12), it is easy to see that the sign of ${\hat{\gamma }}^{\varphi }$ induced by the logistic scaling depends not only on the relative positions of points *P*_0,0_ and *P*_1,0_ (as was the case for monotone convex transformations), but also on their values and the width of the distribution of *ε* (Fig 4A). More specifically, the values of *P*_0,0_ and *P*_1,0_ determine—up to noise—the relation between the magnitudes of A(ε) and B(ε) in (12). Since these values will be different for different genetic variants, the relationship between the signs of ${\hat{\gamma }}^{\varphi }$ and ${\hat{\beta }}^{\varphi }$ can be variant-specific.

Consider an illustrative example of two genetic variants *G*_1_ and *G*_2_. For simplicity, we assume ε=0, which simplifies (11) and (12) to:


$$\begin{array}{cc} & {\hat{\beta }}^{\varphi }=\varphi (P_{0,1})-\varphi (P_{0,0}),\\ & {\hat{\gamma }}^{\varphi }=\varphi (P_{1,1})-\varphi (P_{1,0})-(\varphi (P_{0,1})-\varphi (P_{0,0})). \end{array}$$

For *G*_1_ we assume: $P_{0,0}^{1}=5$, $P_{0,1}^{1}=5.5$, $P_{1,0}^{1}=8$ and $P_{1,1}^{1}=8.5$. This means that in unexposed individuals carrying the reference allele at *G*_1_ the average value of phenotype *Y* is 5, whereas in exposed individuals carrying the same allele it is 8; and the effect of *G*_1_ in both unexposed and exposed groups is 0.5. For variant *G*_2_, on the other hand, we assume: $P_{0,0}^{2}=5.7$, $P_{0,1}^{2}=6.7$, $P_{1,0}^{2}=8.7$ and $P_{1,1}^{2}=9.7$, which corresponds to average values of 5.7 and 8.7 in unexposed and exposed non-carriers, respectively, and the genetic effect of 1 (see x-axis of Fig 4B). Now imagine that we convert phenotype *Y* to a risk scale where the value of 7 corresponds to the risk of 50%: $\varphi (Y)=1/(1+e^{-(Y-7)})$, and perform regression (10). For variant *G*_1_, this regression yields a positive main genetic effect and a positive *G* × *E* effect. For variant *G*_2_, it produces a positive main effect, but a negative *G* × *E* effect (Fig 4B). Thus, in this example the logistic transformation induces *G* × *E* effects that are not sign-consistent.

There are, however, scenarios where the logistic scaling will induce *G* × *E* that are sign-consistent. A plausible example of such a scenario in healthcare data occurs when *x*_0_ in (13) is large (meaning that the cases are called at high phenotype values) and the environmental effect and individual genetic effects on (untransformed) *Y* are relatively small, so that all points *P*_*a*,*b*_ for all considered genetic variants are smaller than *x*_0_. Since the logistic function is convex on the domain $\left(-\inf,x_{0}\right]$, transforming points *P*_*a*,*b*_ with this function yields a relation $sgn({\hat{\gamma }}^{\varphi })=sgn({\hat{\beta }}^{\varphi })$ (Table 1). In general, the sign of a *G* × *E* effect induced by the logistic scaling depends on the relative positions of *P*_0,0_, *P*_1,0_, *P*_0,1_ and *P*_1,1_ with respect to *x*_0_—all possible cases are detailed in S1 File.

#### Case study: the inverse normal transformation.

Another data transformation commonly used in genetic analyses is the INT. It matches quantiles of the data distribution with the quantiles of the standard normal distribution. Because the transformation depends on the data’s distribution, its consequences cannot be generalized. More specifically, INT preserves the order of data points, but not the distances between them. In particular, the relationship between the transformed differences of the conditional means: $P_{0,1}-P_{0,0}$ and $P_{1,1}-P_{1,0}$ (B(ε) and A(ε) in (12)), depends not only on the ordering of these conditional means but also on their magnitudes. Consequently, this relationship need not be the same across variants whose original effects have the same direction. As a result, the INT transformation can induce *G* × *E* effects in any direction with respect to the main genetic effect of a given sign.

### Previously published interaction results exhibit sign consistency property

**Fig 5:**
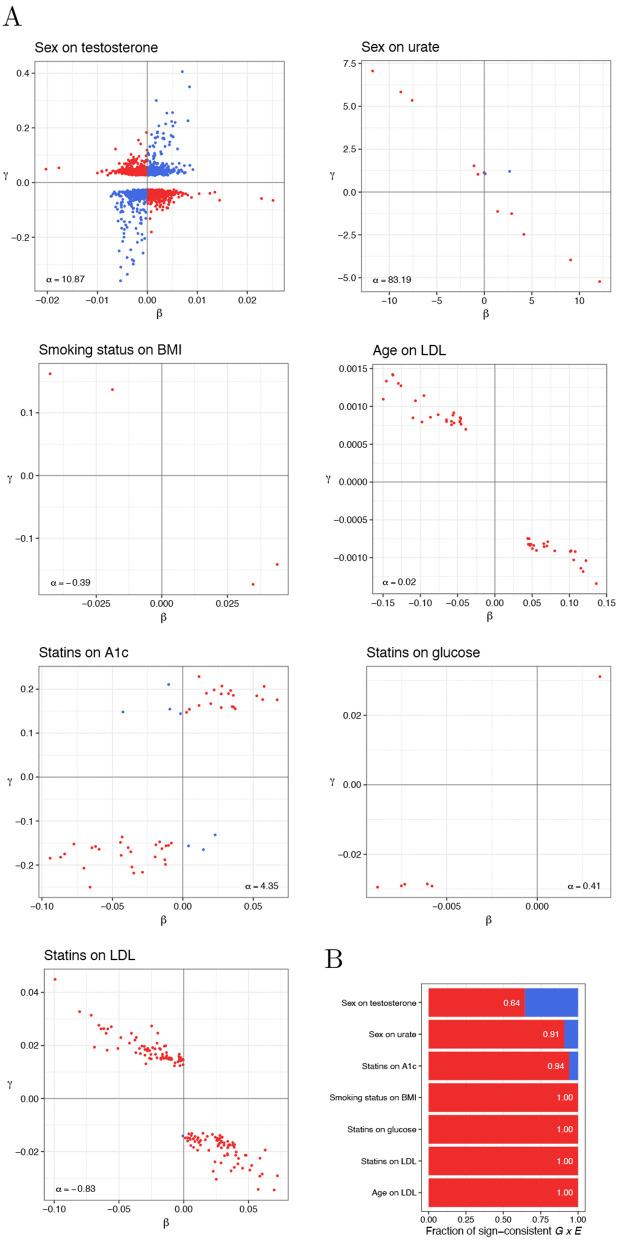
Sign consistency between the main (β) and interaction (γ) effects in TxEWAS for select *E*’s and outcomes. A: Main vs interaction effects for identified genes. For each gene, we plot the estimates corresponding to the tissue with the strongest interaction p-value. α is the main environmental effect. B: The fraction of *G* × *E* that have sign-consistent effects. This fraction was calculated among interacting genes (called at hFDR < 10%) whose main effects were nominally significant at 5%. The tissue with the strongest interaction p-value for a given gene was considered.

We have examined sign consistency for several *G* × *E* studies, selecting *E*-outcome pairs for which interactions have previously been found (Fig 5A). More specifically, we performed TxEWAS [8,27] in the UK Biobank [28] population of unrelated white British individuals (Methods). TxEWAS tests the effect of the interaction between predicted expression of a gene *G* and environmental exposure *E* on phenotype *Y* using the following linear regression model:


$$Y=\mu +\sum_{i}\delta_{i}C_{i}+\sum_{i}\zeta_{i}C_{i}E+\sum_{i}\eta_{i}C_{i}G+\alpha E+\beta G+\gamma GE+\varepsilon ,$$

where *C*_*i*_ is the *i*-th additional environmental covariate included in the model, Greek letters represent effect sizes, and $\varepsilon \sim 𝒩(0,\sigma^{2})$. Among additional covariates we included: age, sex, birth date, Townsend deprivation index, and the first 16 genetic principal components (PCs) [29] (if not already used as *E*). In a single study, we performed multiple tests for a single gene—corresponding to multiple tissues in which this gene was expressed—and used the hierarchical FDR (hFDR) correction to call significant interactions from aggregated results [8]. Sign consistency was examined considering these interactions in tissues, in which they had the strongest effects.

We have investigated gene-sex interaction effects on the primary male sex hormone, testosterone, and the end-product of the purine metabolism, urate; gene-smoking interaction effects on body mass index (BMI); gene-age interaction effects on LDL cholesterol levels; and gene-statin interaction effects on statins’ primary target, LDL cholesterol, and phenotypes related to their potential side effect on diabetes risk [30,31]—blood glucose and hemoglobin A1c (see Methods for phenotype definitions and preprocessing details). We have observed moderate to strong evidence for sign-consistent *G* × *E* effects across these traits. More specifically, the fraction of sign-consistent *G* × *E* effects was moderate for the sex-testosterone *E*-outcome pair, high for the sex-urate and statin-hemoglobin A1c pairs, and maximal for the rest of our studies (Fig 5B).

**Fig 6:**
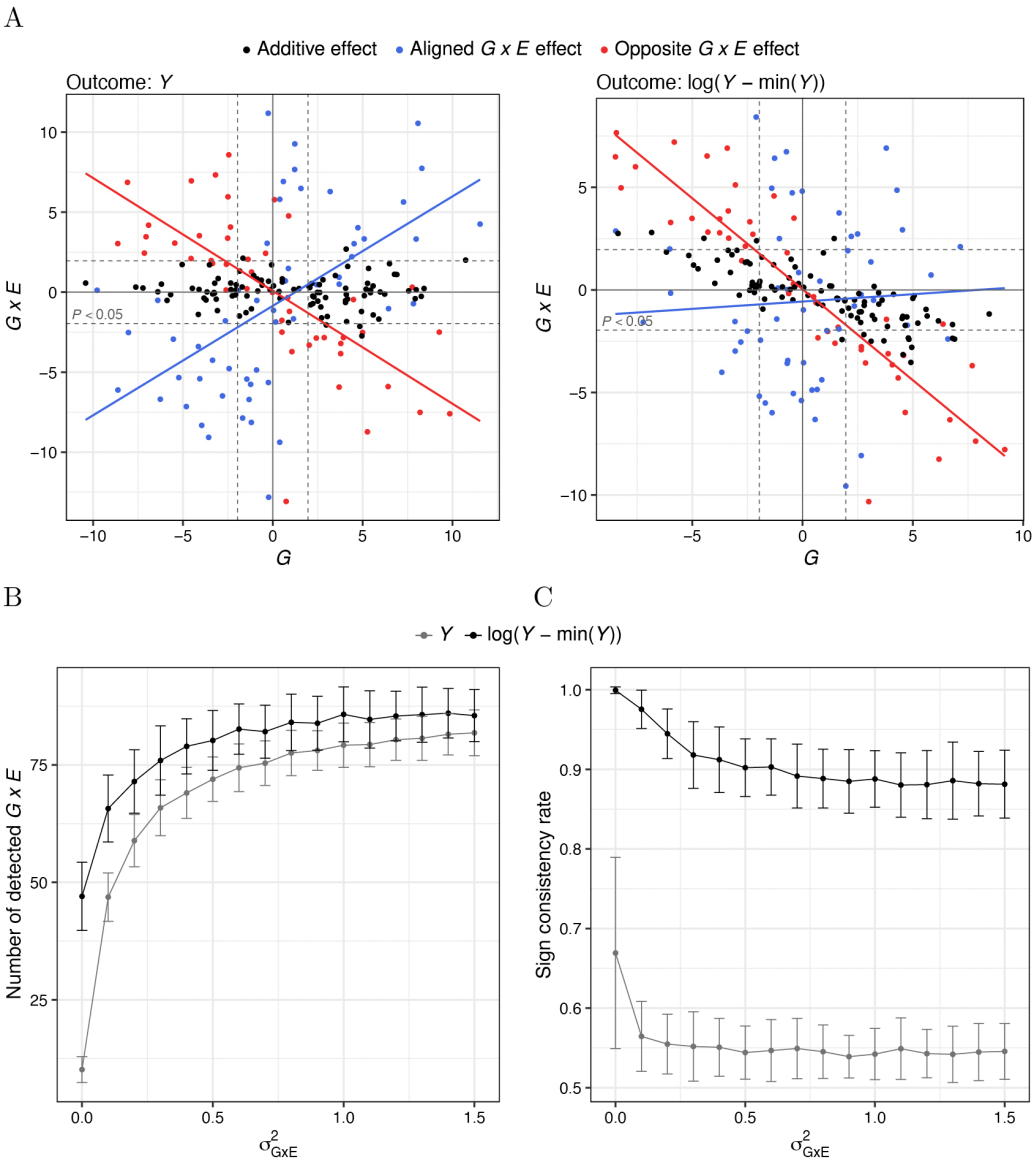
Sign consistency after log-transformation of a simulated outcome with randomly directed G × E effects (Methods; see also S1 and S2 Figs). A: Z-scores for main genetic (*G*) and interaction (*G* × *E*) effects estimated for the outcome before (left) and after (right) transformation. *G* × *E* effects were simulated using $\sigma_{\text{GxE}}^{2}=0.5$. B: Number of detected *G* × *E* effects for outcomes on the original and transformed scales as a function of the variance of the simulated *G* × *E* effects, $\sigma_{\text{GxE}}^{2}$. C: Estimated rate of sign consistency for outcomes on the original and transformed scales as a function of the variance of the simulated *G* × *E* effects, $\sigma_{\text{GxE}}^{2}$. The sign consistency rate was defined as the proportion of *G* × *E* effects exhibiting the more prevalent sign relationship with their corresponding main effects. Due to this definition, sign consistency rate for the untransformed outcome may exceed 0.5.

Such a high degree of sign consistency calls for careful interpretation, as many of these interaction effects may have been induced by the outcome measurement scaling or endogeneity. Monotone convex transformations systematically amplify *G* × *E* effects whose sign is consistent with that of the corresponding main genetic effect, while attenuating interactions with the opposite sign. As a result, the degree of sign consistency after such a transformation can be high even in the presence of *G* × *E* with the opposite sign pattern on the untransformed scale. For example, under an increasing convex up transformation and a positive main environmental effect, *G* × *E* effects opposing the main genetic effects are amplified, whereas those aligned with the genetic main effects are reduced or eliminated (Fig 6A and 6B). Consistent with this intuition, our simulations show high sign consistency rates after applying monotone convex transformations to outcomes with randomly directed *G* × *E* effects (Figs 6 and S1 Fig). Even when the phenotypic variance explained by interaction effects exceeds that of the additive effects, commonly used transformations—such as the logarithm or square—yield sign consistency rates exceeding 75% (Figs 6C and S1B Fig). This rate depends on the directionality and size of the interaction effects on the untransformed scale, and on the specific transformation applied. Consequently, there is no universal threshold that indicates when scaling and endogenous treatment effects should be identified as major drivers of observed *G* × *E* signal. Nevertheless, a predominance of sign-consistent interactions indicates that the results should be interpreted with care and may deserve closer examination. For comparison, our simulations show that the inverse normal transformation, which is not convex, does not alter the sign consistency rate relative to the original scale (S2 Fig).

As a concrete example, we hypothesized that the interaction effects detected in the age-LDL cholesterol study were a consequence of endogenous treatment effects between statin use and LDL cholesterol levels. This is because with age increases the probability of taking statins, which are prescribed at high LDL cholesterol levels—meaning that genetic variation associated with LDL cholesterol levels is also correlated with age. Indeed, when we included statin use in our model as a covariate, the *G* × *E* effects disappeared.

Sign consistency alone cannot determine the extent to which an observed *G* × *E* signal is driven by endogenous treatment effects. For instance, many gene-statin interactions for LDL cholesterol identified by TxEWAS were replicated in a retrospective longitudinal pharmacogenomic study [8], in which major sources of endogeneity were controlled. To determine the mechanisms underpinning such observed associations, additional analyses and experiments are necessary.

## Discussion

We have demonstrated that if there is a scale on which an outcome has homogeneous variance across values of environmental factor *E* and genetic variant *G*, and these factors have only additive effects on this outcome, then the direction of the *G* × *E* effect estimated on the scale that is a monotone convex transformation of the original outcome scale is determined by the direction of the main effect of *G*. In addition, we have shown that endogenous treatment effects, modeled as threshold-based interventions, can only produce *G* × *E* effects with the same sign property.

A consequence of our result is that if *G* × *E* effects in both directions with respect to the main genetic effects are observed, there is no monotone convex transformation that can eliminate the *G* × *E* effects. Furthermore, they could not have been all induced by endogenous treatment effects. Our results are related to prior conditions under which outcome scaling can eliminate interaction effects [20], especially prior results bounding interaction effect sizes as a function of the curvature of the scaling function [32].

Our argument assumes a null interaction effect on some scale to assess the properties of a signal fully attributable to an outcome transformation. Our heuristic examines whether observed interactions are consistent with this hypothesis at a large number of loci. Although it is unreasonable in general to imagine that all variants interact in the same way relative to an environmental moderator, a monotone convex transformation of the outcome results in a high sign consistency rate even if this null hypothesis is not true for every locus. Thus, a predominance of sign-consistent interactions provides a meaningful indication that the results should be interpreted with care and may deserve closer examination.

Despite apparent similarities between endogenous treatment effects and gene-environment (*G*-*E*) correlation, the two phenomena differ. In the considered model of outcome-dependent treatment allocation, genetic factors that influence the outcome become associated with treatment status, and, critically, treatment status becomes correlated with the error term. A correlation between genetic factors and exposure alone does not induce statistical *G* × *E* effects. Although *G*-*E* correlation can produce spurious *G* × *E* signals when genetic markers such as tag SNPs are analyzed instead of the true causal variants [33], this arises from a different data-generating process and is likely a much weaker source of misleading *G* × *E* findings [34].

Sign consistency of *G* × *E* effects does not imply that they are induced by endogenous treatment effects, nor does the fact that they can be eliminated by an outcome transformation imply that the outcome should be analyzed on the transformed scale. Whenever possible, the outcome scale should be chosen to ensure that results are interpretable and practically meaningful. For example, the relevant scale may be determined by the specific mechanistic model of a biological phenomenon under study or by the public health intervention being evaluated. However, as it is generally unclear what the *correct* scale is for a given phenotype, examining sign consistency across observed *G* × *E* effects can help assess the extent to which a particular type of outcome transformation may alter the results. Moreover, such examination can rule out the possibility that all observed interactions can be attributed to endogenous treatment effects in studies where such effects may be present and the underlying causal mechanisms are unknown. Our analysis of real data sets demonstrates that our approach can help identify potential confounding.

To reduce inaccuracy in assessing sign consistency, we recommend applying the sign consistency property to genome-wide significant interactions (or, at a minimum, to a threshold determined *a priori*), as done in the analyses presented in this paper.

We note that the homoskedasticity assumption made in our proofs is also an assumption of the linear regression model. Violation of this assumption results in a biased test for the interaction effect [8,35]. In the observed data, it is specifically common that the variance of the outcome differs across strata defined by the environmental factor [36]. Owing to its importance and incomplete characterization, we comprehensively examine the conditional heteroskedasticity bias in S1 Supporting Information. We analytically describe the conditions under which this bias is expected to arise and the direction of its effect. It has been established that, in the presence of heteroskedasticity, *G* × *E* should be modeled using the double generalized linear model or a standard linear model modified to incorporate robust standard errors [8,35].

## Methods

### Sign consistency of *G* × *E* effects under monotone convex transformations of the outcome

Here we provide geometric intuition for the sign-consistent interaction property; a direct algebraic derivation is given in S1 Appendix.

Suppose that there is a scale, on which a phenotype exhibits no *G* × *E*, and has homogeneous variance. We show that any monotone convex transformation of this phenotype can only induce sign-consistent *G* × *E* effects (Theorem 1). The implication is that if *G* × *E* effects in both directions with respect to the main genetic effects are observed, there is no such transformation that can eliminate the *G* × *E* effects.

Specifically, consider phenotype *Y* that has homogeneous variance across values of binary environmental factor *E* and haploid genotype *G*:


$$\forall_{a,b\in \{0,1\}}Y{|}_{E=a,G=b}\sim 𝒟(\nu_{a,b},\sigma^{2}),$$

where 𝒟 is a symmetric distribution with mean *ν* and variance $\sigma^{2}$. Consider further fitting the following linear regression model to *Y*:


$$Y=\hat{\mu }+\hat{\alpha }E+\hat{\beta }G+\hat{\gamma }GE+\varepsilon ,$$ (14)

where $\hat{\mu }$, $\hat{\alpha }$, $\hat{\beta }$ and $\hat{\gamma }$ are estimated coefficients, and *ε* is the error. We assume that:


$$\hat{\gamma }=0.$$

The coefficients in (14) can be related to the empirical conditional means of *Y*, which we denote by points $P_{a,b}:=\hat{𝔼}[Y|E=a,G=b]$:


$$\begin{array}{cc} & \hat{\mu }=P_{0,0},\\ & \hat{\alpha }=P_{1,0}-P_{0,0},\\ & \hat{\beta }=P_{0,1}-P_{0,0},\\ & \hat{\gamma }=(P_{1,1}-P_{1,0})-(P_{0,1}-P_{0,0}). \end{array}$$

**Fig 7:**
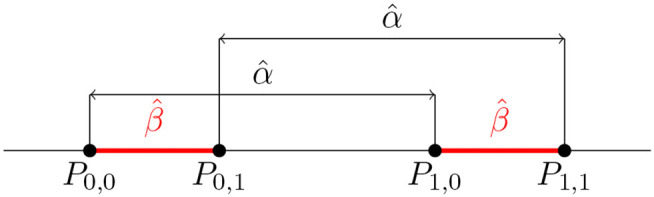
The order of points *P*_*a*,*b*_. When $\hat{\gamma }=0$, the signs of regression coefficients $\hat{\alpha }$ and $\hat{\beta }$ in (14) determine the order of *P*_0,0_ and *P*_0,1_, *P*_1,0_ and *P*_1,1_, *P*_0,0_ and *P*_1,0_, and *P*_0,1_ and *P*_1,1_. Here, $0<\hat{\beta }<\hat{\alpha }$.

The order of *P*_0,0_ and *P*_0,1_, *P*_1,0_ and *P*_1,1_, *P*_0,0_ and *P*_1,0_, and *P*_0,1_ and *P*_1,1_ is determined by the signs of coefficients $\hat{\beta }$ and $\hat{\alpha }$. To see this, note that if $\hat{\beta }>0$, then $P_{0,0}<P_{0,1}$. Alternatively, if $\hat{\beta }<0$, then $P_{0,0}>P_{0,1}$. Furthermore, by the assumption that $\hat{\gamma }$ is null, $P_{1,0}=P_{0,0}+\hat{\alpha }$ and $P_{1,1}=P_{0,1}+\hat{\alpha }$ (Fig 7).

We will use this fact to show that a regression similar to (14) on a monotone convex transformation of *Y* yields *G* × *E* effects whose directions depend on the directions of the corresponding main genetic effects.

Consider *Y* transformed by a function φ, and a linear regression of this transformed *Y* on *E*, *G* and *GE*:


$$\varphi (Y)={\hat{\mu }}^{\varphi }+{\hat{\alpha }}^{\varphi }E+{\hat{\beta }}^{\varphi }G+{\hat{\gamma }}^{\varphi }GE+\varepsilon^{\varphi }.$$ (15)

We can relate the coefficients ${\hat{\beta }}^{\varphi }$ in (15) to points *P*_0,0_, *P*_0,1_, *P*_1,0_, and *P*_1,1_ that we have defined on the original scale of *Y*:


$$\begin{array}{c} \begin{array}{cc} {\hat{\beta }}^{\varphi } & =\hat{𝔼}[\varphi (Y)|E=0,G=1]-\hat{𝔼}[\varphi (Y)|E=0,G=0]=\hat{𝔼}[\varphi (P_{0,1}+\varepsilon )-\varphi (P_{0,0}+\varepsilon )]\\ & =\int [\varphi (P_{0,1}+\varepsilon )-\varphi (P_{0,0}+\varepsilon )]f(\varepsilon )d\varepsilon , \end{array} \end{array}$$

and likewise for ${\hat{\gamma }}^{\varphi }$:


$$\begin{array}{c} \begin{array}{cc} {\hat{\gamma }}^{\varphi } & =\hat{𝔼}[\varphi (Y)|E=1,G=1]-\hat{𝔼}[\varphi (Y)|E=1,G=0]-(\hat{𝔼}[\varphi (Y)|E=0,G=1]-\hat{𝔼}[\varphi (Y)|E=0,G=0])\\ & =\hat{𝔼}[\varphi (P_{1,1}+\varepsilon )-\varphi (P_{1,0}+\varepsilon )-(\varphi (P_{0,1}+\varepsilon )-\varphi (P_{0,0}+\varepsilon ))]\\ & =\int [\varphi (P_{1,1}+\varepsilon )-\varphi (P_{1,0}+\varepsilon )-(\varphi (P_{0,1}+\varepsilon )-\varphi (P_{0,0}+\varepsilon ))]f(\varepsilon )d\varepsilon , \end{array} \end{array}$$

where *f* is the PDF of *ε*. Note that each point *P*_*a*,*b*_ above is always shifted by the same value; and that the signs of the above expressions are invariant to this shift if φ is monotone convex:


$$sgn({\hat{\beta }}^{\varphi })=sgn(\varphi (P_{0,1})-\varphi (P_{0,0})),$$ (16)

$$sgn({\hat{\gamma }}^{\varphi })=sgn(\varphi (P_{1,1})-\varphi (P_{1,0})-(\varphi (P_{0,1})-\varphi (P_{0,0}))).$$ (17)

**Fig 8:**
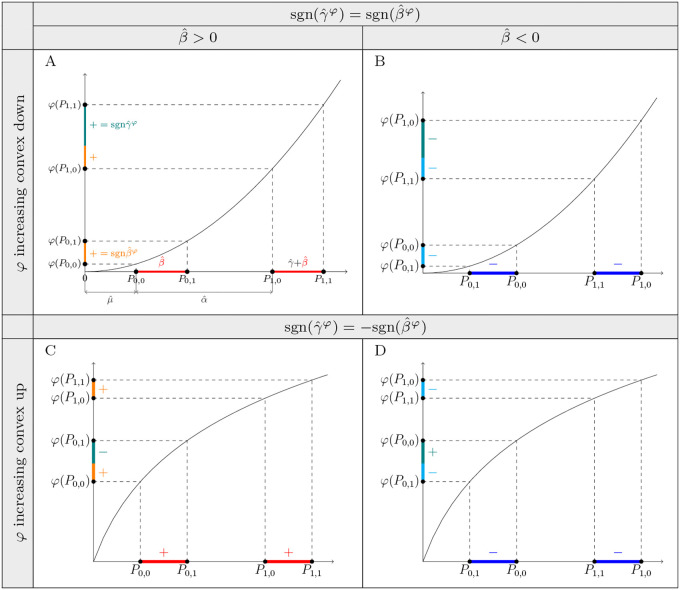
Increasing convex transformations of the outcome induce *G* × *E* effects, ${\hat{\gamma }}^{\varphi }$, whose direction is determined by the direction of the main genetic effects, ${\hat{\beta }}^{\varphi }$, if the untransformed outcome exhibits no *G* × *E* and has homogeneous variance in regression (14). A: The relation between the signs of ${\hat{\gamma }}^{\varphi }$ and ${\hat{\beta }}^{\varphi }$ when $\hat{\beta }$ and $\hat{\alpha }$ are positive and φ is increasing convex down. B: Similar to A, but when $\hat{\beta }$ is negative. C: Similar to A, but when φ is increasing convex up. D: Similar to A, but when $\hat{\beta }$ is negative and φ is increasing convex up.

Without loss of generality, suppose that φ is increasing convex down. To determine the sign of ${\hat{\gamma }}^{\varphi }$, we need to know the signs of differences $\varphi (P_{0,1})-\varphi (P_{0,0})$ and $\varphi (P_{1,1})-\varphi (P_{1,0})$, and the relation between their magnitudes. Since *P*_1,0_ and *P*_1,1_ are shifted from *P*_0,0_ and *P*_0,1_ by the same value, $\hat{\alpha }$, the signs of $\varphi (P_{0,1})-\varphi (P_{0,0})$ and $\varphi (P_{1,1})-\varphi (P_{1,0})$ are the same, and, by (16), follow the sign of ${\hat{\beta }}^{\varphi }$ (Fig 8A). The relation between their magnitudes depends on the sign of $\hat{\alpha }$. If $\hat{\alpha }$ is positive, the magnitude of $\varphi (P_{1,1})-\varphi (P_{1,0})$ is greater than the magnitude of $\varphi (P_{0,1})-\varphi (P_{0,0})$, and the opposite is true if $\hat{\alpha }$ is negative.

If two genetic variants, *G*_1_ and *G*_2_, are regressed like *G* in (15)—and the assumptions of model (14) are met—such that the sign of $\hat{\alpha }$ in these two cases is the same, the sign of ${\hat{\gamma }}^{\varphi }$ differs between these regressions only if the sign of ${\hat{\beta }}^{\varphi }$ differs.

For example, when φ is increasing convex down and $\hat{\alpha }$ is positive, then:

- ${\hat{\beta }}^{\varphi }>0$ implies ${\hat{\gamma }}^{\varphi }>0$, because both $\varphi (P_{0,1})-\varphi (P_{0,0})$ and $\varphi (P_{1,1})-\varphi (P_{1,0})$ are positive, and $\left|\varphi (P_{0,1})-\varphi (P_{0,0})\right|<\left|\varphi (P_{1,1})-\varphi (P_{1,0})\right|$ (Fig 8A).
- ${\hat{\beta }}^{\varphi }<0$ implies ${\hat{\gamma }}^{\varphi }<0$, because both $\varphi (P_{0,1})-\varphi (P_{0,0})$ and $\varphi (P_{1,1})-\varphi (P_{1,0})$ are negative, and $\left|\varphi (P_{0,1})-\varphi (P_{0,0})\right|<\left|\varphi (P_{1,1})-\varphi (P_{1,0})\right|$ (Fig 8B).

Therefore, in this example, $sgn({\hat{\gamma }}^{\varphi })=sgn({\hat{\beta }}^{\varphi })$.

Similarly, when $\hat{\alpha }$ is positive, but φ is increasing convex up, then:

- ${\hat{\beta }}^{\varphi }>0$ implies ${\hat{\gamma }}^{\varphi }<0$, because both $\varphi (P_{0,1})-\varphi (P_{0,0})$ and $\varphi (P_{1,1})-\varphi (P_{1,0})$ are positive, and $\left|\varphi (P_{0,1})-\varphi (P_{0,0})\right|>\left|\varphi (P_{1,1})-\varphi (P_{1,0})\right|$ (Fig 8C).
- ${\hat{\beta }}^{\varphi }<0$ implies ${\hat{\gamma }}^{\varphi }>0$, because both $\varphi (P_{0,1})-\varphi (P_{0,0})$ and $\varphi (P_{1,1})-\varphi (P_{1,0})$ are negative, and $\left|\varphi (P_{0,1})-\varphi (P_{0,0})\right|>\left|\varphi (P_{1,1})-\varphi (P_{1,0})\right|$ (Fig 8D).

Therefore, in this example, $sgn({\hat{\gamma }}^{\varphi })=-sgn({\hat{\beta }}^{\varphi })$.

Note that if φ is increasing convex down, −φ is decreasing convex up; and if φ is increasing convex up, −φ is decreasing convex down. Change of the sign of the function inverts the directions of both ${\hat{\gamma }}^{\varphi }$ and ${\hat{\beta }}^{\varphi }$ (see (16) and (17)). Thus, those pairs of transformations, induce the same relation between the signs of ${\hat{\gamma }}^{\varphi }$ and ${\hat{\beta }}^{\varphi }$.

Finally, change of the sign of $\hat{\alpha }$, inverts the relation between the magnitudes of differences $\varphi (P_{0,1})-\varphi (P_{0,0})$ and $\varphi (P_{1,1})-\varphi (P_{1,0})$, which, for a given transformation, results in an inverted relation between the signs of ${\hat{\gamma }}^{\varphi }$ and ${\hat{\beta }}^{\varphi }$. We summarize all possible cases in Table 2.

**Table 2:** Monotone convex transformations induce *G* × *E* effects (${\hat{\gamma }}^{\varphi }$) whose directions are consistent with the directions of the observed main effects (${\hat{\beta }}^{\varphi }$).

	Example functions	$\hat{\alpha }>0$	$\hat{\alpha }<0$
φ increasing convex down	$\varphi (x)=x^{2}\;\text{for}\;x\geq 0$, $\varphi (x)=e^{x}$	$\begin{array}{c} sgn({\hat{\gamma }}^{\varphi })=sgn({\hat{\beta }}^{\varphi }) \end{array}$	$\begin{array}{c} sgn({\hat{\gamma }}^{\varphi })=-sgn({\hat{\beta }}^{\varphi }) \end{array}$
φ decreasing convex down	$\varphi (x)=x^{2}\;\text{for}\;x\leq 0$, $\varphi (x)=e^{-x}$	$\begin{array}{c} sgn({\hat{\gamma }}^{\varphi })=-sgn({\hat{\beta }}^{\varphi }) \end{array}$	$\begin{array}{c} sgn({\hat{\gamma }}^{\varphi })=sgn({\hat{\beta }}^{\varphi }) \end{array}$
φ increasing convex up	$\varphi (x)=\sqrt{x}\;\text{for}\;x\geq 0$, φ(x)=log(x)	$\begin{array}{c} sgn({\hat{\gamma }}^{\varphi })=-sgn({\hat{\beta }}^{\varphi }) \end{array}$	$\begin{array}{c} sgn({\hat{\gamma }}^{\varphi })=sgn({\hat{\beta }}^{\varphi }) \end{array}$
φ decreasing convex up	$\varphi (x)=-x^{2}\;\text{for}\;x\geq 0$, $\varphi (x)=-e^{x}$	$\begin{array}{c} sgn({\hat{\gamma }}^{\varphi })=sgn({\hat{\beta }}^{\varphi }) \end{array}$	$\begin{array}{c} sgn({\hat{\gamma }}^{\varphi })=-sgn({\hat{\beta }}^{\varphi }) \end{array}$

In S1 Appendix, we show that the distinction between increasing and decreasing transformations is absorbed into the observed coefficients ${\hat{\alpha }}^{\varphi }$ and ${\hat{\beta }}^{\varphi }$, yielding the unified sign rule $sgn({\hat{\gamma }}^{\varphi })=sgn(\varphi^{\prime \prime })\cdot sgn({\hat{\alpha }}^{\varphi })\cdot sgn({\hat{\beta }}^{\varphi })$. In practice, this formula can be applied directly using the estimated coefficients without needing to determine whether the transformation is increasing or decreasing.

### Sign consistency of *G* × *E* effects under a threshold-based model of endogenous treatment effects

Consider the following model of phenotype *Y*:


$$\begin{array}{cc} & Y=\sum_{i}\beta_{i}G_{i}+\varepsilon ,\\ & G_{i}\sim \text{Bernoulli}(p_{i}),\\ & \varepsilon \sim 𝒩(0,\sigma^{2}), \end{array}$$

where *G*_*i*_ indicates the presence of an alternative allele at variant *i*, $\beta_{i}$ is the effect of this allele on *Y*, and *ε* is the environmental noise. We assume that the genotypes are independent: $\forall_{i\neq j}G_{i}⟂\;\;\;⟂G_{j}$, and that the environmental noise is homoskedastic: $\forall_{i}Var[\varepsilon |G_{i}=0]=Var[\varepsilon |G_{i}=1]$. Note that, unlike in our previous derivation where no specific generating model is assumed, here we assume this is the actual generating process. Without loss of generality, let $\sigma^{2}=1$.

Suppose that if the level of *Y* is high, treatment *E* is administered:


$$\begin{array}{c} E=\left\{ \begin{array}{cc} 1 & \text{if }Y>t,\\ 0 & \text{otherwise}, \end{array}\right. \end{array}$$

where *t* is some threshold. When applied, treatment *E* changes the level of *Y* by $\begin{array}{c} \alpha \end{array}$:


$$\tilde{Y}=Y+\alpha E.$$

Suppose further that we observe phenotype $\tilde{Y}$ and test the effect ${\hat{\gamma }}_{j}$ of the interaction between variant *G*_*j*_ and environmental factor *E* on this phenotype:


$$\tilde{Y}={\hat{\mu }}_{j}+{\hat{\alpha }}_{j}E+{\hat{\beta }}_{j}G_{j}+{\hat{\gamma }}_{j}G_{j}E+\varepsilon_{j}.$$ (18)

We prove that the sign of ${\hat{\gamma }}_{j}$ is determined by the sign of the main effect ${\hat{\beta }}_{j}$ of *G*_*j*_ (Claim 1).

Note that coefficients ${\hat{\beta }}_{j}$ and ${\hat{\gamma }}_{j}$ can be related to empirical conditional expectations of $\tilde{Y}$:


$$\begin{array}{cc} & {\hat{\beta }}_{j}=\hat{𝔼}[\tilde{Y}|E=0,G_{j}=1]-\hat{𝔼}[\tilde{Y}|E=0,G_{j}=0],\\ & {\hat{\gamma }}_{j}=\hat{𝔼}[\tilde{Y}|E=1,G_{j}=1]-\hat{𝔼}[\tilde{Y}|E=1,G_{j}=0]-(\hat{𝔼}[\tilde{Y}|E=0,G_{j}=1]-\hat{𝔼}[\tilde{Y}|E=0,G_{j}=0]). \end{array}$$

Furthermore, note that phenotype $\tilde{Y}$ conditioned on the value of *E* has a truncated normal distribution, and its conditional mean is given by:


$$\begin{array}{cc} & 𝔼[\tilde{Y}|E=0]=\mu -\frac{\phi (t-\mu )}{\Phi (t-\mu )},\\ & 𝔼[\tilde{Y}|E=1]=\mu +\frac{\phi (t-\mu )}{1-\Phi (t-\mu )}+\alpha , \end{array}$$

where φ is the probability density function and Φ is the cumulative distribution function of the standard normal distribution, and μ is the mean of *Y*. Furthermore, the value of μ depends on the genotype *G*_*j*_:


$$\begin{array}{cc} & 𝔼[\tilde{Y}|E=0,G_{j}=0]=\mu_{j}^{0}-\frac{\phi (t-\mu_{j}^{0})}{\Phi (t-\mu_{j}^{0})},\\ & 𝔼[\tilde{Y}|E=0,G_{j}=1]=\mu_{j}^{1}-\frac{\phi (t-\mu_{j}^{1})}{\Phi (t-\mu_{j}^{1})},\\ & 𝔼[\tilde{Y}|E=1,G_{j}=0]=\mu_{j}^{0}+\frac{\phi (t-\mu_{j}^{0})}{1-\Phi (t-\mu_{j}^{0})}+\alpha ,\\ & 𝔼[\tilde{Y}|E=1,G_{j}=1]=\mu_{j}^{1}+\frac{\phi (t-\mu_{j}^{1})}{1-\Phi (t-\mu_{j}^{1})}+\alpha , \end{array}$$

where $\mu_{j}^{a}:=𝔼[Y|G_{j}=a]$.

To simplify the above expressions, we denote the inverse Mill’s Ratio λ(x):=ϕ(x)/Φ(x), and note that ϕ(x)/(1−Φ(x))=ϕ(−x)/Φ(−x)=λ(−x), because φ is even, and Φ(−x)=1−Φ(x). Furthermore, we define points $P_{0}:=t-\mu_{j}^{0}$ and $\begin{array}{c} P_{1}:=t-\mu_{j}^{1} \end{array}$, and express the estimated effects ${\hat{\beta }}_{j}$ and ${\hat{\gamma }}_{j}$ in (18) as a function of these points:


$${\hat{\beta }}_{j}=\mu_{j}^{1}-\lambda (P_{1})-\mu_{j}^{0}+\lambda (P_{0})=\lambda (P_{0})-\lambda (P_{1})+P_{0}-P_{1},$$ (19)

$$\begin{array}{c} \begin{array}{cc} {\hat{\gamma }}_{j} & =\mu_{j}^{1}+\lambda (-P_{1})+\alpha -\mu_{j}^{0}-\lambda (-P_{0})-\alpha -(\mu_{j}^{1}-\lambda (P_{1})-\mu_{j}^{0}+\lambda (P_{0}))\\ & =\lambda (-P_{1})-\lambda (-P_{0})-(\lambda (P_{0})-\lambda (P_{1})). \end{array} \end{array}$$ (20)

**Fig 9:**
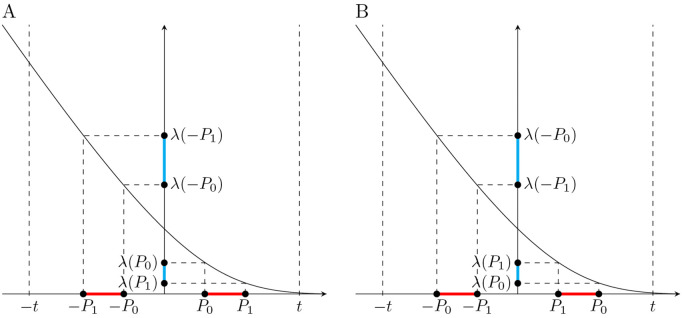
Endogenous treatment effects induce sign-consistent *G* × *E* effects. A: The x-axis shows the quantities $P_{0}:=t-\mu_{j}^{0}$ and $P_{1}:=t-\mu_{j}^{1}$ defined for phenotype *Y* affected by the haploid genetic variant *G*_*j*_, where $0<\mu_{j}^{1}<\mu_{j}^{0}<t$. The main effect of *G*_*j*_ and the effect of *G*_*j*_*E* on phenotype *Y*, after treatment *E* is applied to reduce levels of *Y* that exceed threshold *t*, can be expressed as functions of *P*_0_ and *P*_1_ and their images under the inverse Mill’s Ratio $\lambda (P_{0})$ and $\lambda (P_{1})$. The signs of those functions are dependent. B: Similar to A, but when $0<\mu_{j}^{0}<\mu_{j}^{1}<t$.

The function Φ is decreasing and strictly convex down [26] (Fig 9). As a result, the order of points *P*_0_ and *P*_1_ determines the signs of ${\hat{\beta }}_{j}$ and ${\hat{\gamma }}_{j}$. Without loss of generality, let *t* > 0. Since *t* distinguishes “high” from “normal” levels of phenotype *Y*, it is reasonable to assume that means $\mu_{j}^{0}$ and $\mu_{j}^{1}$ are smaller than *t* (note that variants *G*_*i*_ are independent); that is, any individual SNP does not result in high enough *Y* to receive the treatment, as it is likely for any polygenic trait. There are therefore two possible cases:

1. $t>\mu_{j}^{0}>\mu_{j}^{1}$, which imposes the following order on the points used in definitions (19) and (20): $-P_{1}<-P_{0}<P_{0}<P_{1}$ (Fig 9A). Given this order and the properties of λ, we have: 1) $P_{0}-P_{1}<0$, $\lambda (P_{0})-\lambda (P_{1})>0$, and $\left|\lambda (P_{0})-\lambda (P_{1})\right|<\left|P_{0}-P_{1}\right|$, which implies that ${\hat{\beta }}_{j}$ is negative; and 2) $0<\lambda (P_{0})-\lambda (P_{1})<\lambda (-P_{1})-\lambda (-P_{0})$, which implies that ${\hat{\gamma }}_{j}$ is positive.
2. $t>\mu_{j}^{1}>\mu_{j}^{0}$, which results in: $-P_{0}<-P_{1}<P_{1}<P_{0}$ (Fig 9B). Given this order and the properties of λ, we have: 1) $P_{0}-P_{1}>0$, $\lambda (P_{0})-\lambda (P_{1})<0$, and $\left|\lambda (P_{0})-\lambda (P_{1})\right|<\left|P_{0}-P_{1}\right|$, which implies that ${\hat{\beta }}_{j}$ is positive; 2) $\lambda (-P_{1})-\lambda (-P_{0})<\lambda (P_{0})-\lambda (P_{1})<0$, which implies that ${\hat{\gamma }}_{j}$ is negative.

We have, therefore, shown that:


$$sgn({\hat{\gamma }}_{j})=-sgn({\hat{\beta }}_{j}),$$ (21)

which means that the estimated effects ${\hat{\beta }}_{j}$ and ${\hat{\gamma }}_{j}$ in regression (18) have opposite directions. It can be analogously shown that relation (21) holds when treatment *E* is administered whenever the level of phenotype *Y* is below threshold *t*.

### Simulation details

We conducted simulations to assess sign consistency of estimated *G* × *E* effects after applying monotone convex transformations to outcomes that exhibit *G* × *E* on the original scale. In each simulation, outcomes were generated according to $Y\sim 𝒩(2E+X\beta +(X*E)\gamma ,\sigma_{\varepsilon }^{2})$, where *E* is a binary environmental exposure, *X* is a matrix of 200 independent diploid SNPs, and X*E denotes the element-wise product of *E* with each column of *X*. Additive genetic effects β were drawn from a standard normal distribution. We randomly selected 100 SNPs to also have interaction effects, with half having interaction effects aligned in sign and half opposed in sign relative to their corresponding main effects. Interaction effects γ for these 100 SNPs were drawn from a Gaussian distribution with mean zero and variance $\sigma_{\text{GxE}}^{2}$. The residual variance $\sigma_{\varepsilon }^{2}$ was scaled to achieve heritability 0.5 among samples with *E* = 0. A total of 10,000 samples were simulated (5,000 per environmental condition). Statistical significance of *G* × *E* effects was assessed at a 5% false positive rate using single-SNP regressions applied to the original outcome *Y* and to the transformed outcome.

To estimate the number of detected *G* × *E* effects and the sign consistency rate, 100 independent replicates were performed for each value of $\sigma_{\text{GxE}}^{2}$, and results were summarized using the mean and standard deviation.

### TxEWAS in the UK Biobank

The TxEWAS presented in this work were performed following the Sadowski et al. protocol [27]. The studied UK Biobank population of 342,257 unrelated white British individuals was identified by performing the steps described by Sadowski et al. [8]

We imputed gene expression into the UK Biobank using eQTL weights trained in 48 tissues of The Genotype-Tissue Expression (GTEx v7) project, linked by the TxEWAS protocol [27]. Hierarchical FDR (hFDR < 10%) was used to account for multiple hypothesis testing across genes and tissues [37,38].

Individuals who took statins were identified by codes: 1140861958, 1140861970, 1141146138, 1140888594, 1140888648, 1140910632, 1140910654, 1141146234, 1141192410, 1141192414, 1141188146, 1140881748, and 1140864592 in the UK Biobank field 20003-0.0-47. Smoking status was derived from the UK Biobank field 20116-0.0 by encoding the “current” category as 1, and the categories of “never” and “previous” as 0.

For all tested outcomes except testosterone, we discarded measurements greater than five standard deviations from the mean, with the assumption that such extreme levels were results of non-modeled circumstances. The distribution of testosterone levels was bimodal, but the sign consistency pattern for this phenotype presented in Fig 5A remained similar after inverse normally transforming it.

We included age, sex, birth date, Townsend deprivation index, and the first 16 genetic PCs [29] as covariates in our studies. All non-binary covariates were standardized (transformed to mean zero, variance one) before calculating interaction variables.

## Supporting information

S1 Appendix Derivation of the sign-consistent interaction property. (PDF)

S1 File Supporting Information. Supplementary notes.
(PDF)

S1 FigSign consistency after square-transformation of a simulated outcome with randomly directed *G* × *E* effects (Methods).A: Number of detected *G* × *E* effects for outcomes on the original and transformed scales as a function of the variance of the simulated *G* × *E* effects, $\sigma_{\text{GxE}}^{2}$. B: Estimated rate of sign consistency for outcomes on the original and transformed scales as a function of the variance of the simulated *G* × *E* effects, $\sigma_{\text{GxE}}^{2}$. The sign consistency rate was defined as the proportion of *G* × *E* effects exhibiting the more prevalent sign relationship with their corresponding main effects. Due to this definition, sign consistency rate for the untransformed outcome may exceed 0.5.(PDF)

S2 FigSign consistency after inverse normal transformation of a simulated outcome with randomly directed *G* × *E* effects (Methods).A: Number of detected *G* × *E* effects for outcomes on the original and transformed scales as a function of the variance of the simulated *G* × *E* effects, $\sigma_{\text{GxE}}^{2}$. B: Estimated rate of sign consistency for outcomes on the original and transformed scales as a function of the variance of the simulated *G* × *E* effects, $\sigma_{\text{GxE}}^{2}$. The sign consistency rate was defined as the proportion of *G* × *E* effects exhibiting the more prevalent sign relationship with their corresponding main effects. Due to this definition, sign consistency rate for the untransformed outcome may exceed 0.5.(PDF)
