# Supplementary material for: The geometry of G × E: How scaling and endogenous treatment effects shape interaction direction
Source: PLoS Genet. 2026 Apr 1;22(4):e1012073. doi: 10.1371/journal.pgen.1012073 (PMC13043064; doi:10.1371/journal.pgen.1012073)
Supplement: S1 Appendix — (PDF) [file pgen.1012073.s001.pdf]

S1 Appendix

The geometry of $G \times E$: how scaling and endogenous treatment effects shape interaction direction

Michal Sadowski, Andy W. Dahl, Noah Zaitlen, and Richard Border

Here we provide complete proofs of the theorems and corollaries stated in the main manuscript. Section 1 establishes the sign rule for transformation-induced interactions when G and E are independent (Theorem 1, Corollaries 1–2). Section 2 extends these results to allow gene-environment correlation (Theorem 2, Corollary 3). Section 3 proves the opposite-sign rule for endogenous treatment effects (Theorem 3–4).

Beyond the qualitative sign rules, we also quantify the *magnitude* of the induced interactions. We introduce two scale-free nonlinearity coefficients: κ for scaling effects and η for endogenous treatment effects. These coefficients capture the strength of the induced interaction relative to the main effects, enabling quantitative predictions beyond the directional sign rule.

1 Transformations with independent G and E

We begin with the case where genotype G and environment E are independent random variables. The following assumptions are maintained throughout (with the exception of A2, which we later relax):

(A1) **Centered predictors:** $\mathbb{E}[G] = \mathbb{E}[E] = 0$.

(A2) **Independence:** $G \perp\!\!\!\perp E$.

(A3) **Finite moments:** $\text{Var}(G), \text{Var}(E) \in (0, \infty)$.

(A4) **Residual independence:** $\varepsilon \perp\!\!\!\perp (G, E)$.

(A5) **Residual regularity:** $\mathbb{E}[\varepsilon] = 0$ and $\text{Var}(\varepsilon) = \sigma_\varepsilon^2 < \infty$.

(A6) **Transformation smoothness:** $\varphi : \mathbb{R} \to \mathbb{R}$ is twice-differentiable with $\mathbb{E}[|\varphi''(\varepsilon)|] < \infty$.

Third moments $\mathbb{E}[G^3]$ and $\mathbb{E}[E^3]$ appear in correction terms but vanish for symmetric distributions, which simplifies several results.

Consider a phenotype Y generated by an additive model with no interaction:

$$Y = \alpha E + \beta G + \varepsilon,$$

where $\alpha, \beta \in \mathbb{R}$ are the main effects for environment and genotype respectively. The residual ε is independent of (G, E), has zero mean, and finite variance σ_ε^2. These assumptions ensure homoskedasticity on the original scale: $\mathrm{Var}(Y|G, E) = \sigma_\varepsilon^2$ for all (G, E).

After applying a twice-differentiable transformation $\varphi : \mathbb{R} \to \mathbb{R}$, we fit:

$$\varphi(Y) = \hat{\mu}^\varphi + \hat{\alpha}^\varphi E + \hat{\beta}^\varphi G + \hat{\gamma}^\varphi GE + \varepsilon^\varphi.$$

Under assumptions (A1)–(A2), the centered predictors G, E, and GE are mutually orthogonal:

$$\mathrm{Cov}(G, E) = \mathrm{Cov}(G, GE) = \mathrm{Cov}(E, GE) = 0.$$

Therefore, the OLS coefficients decompose as simple projections:

$$\hat{\alpha}^\varphi = \frac{\mathrm{Cov}(E, \varphi(Y))}{\mathrm{Var}(E)} = \frac{\mathbb{E}[E \cdot \varphi(Y)]}{\mathrm{Var}(E)}, \tag{1}$$

$$\hat{\beta}^\varphi = \frac{\mathrm{Cov}(G, \varphi(Y))}{\mathrm{Var}(G)} = \frac{\mathbb{E}[G \cdot \varphi(Y)]}{\mathrm{Var}(G)},$$

$$\hat{\gamma}^\varphi = \frac{\mathrm{Cov}(GE, \varphi(Y))}{\mathrm{Var}(GE)} = \frac{\mathbb{E}[GE \cdot \varphi(Y)]}{\mathrm{Var}(G)\mathrm{Var}(E)}, \tag{2}$$

where the last equality uses $\mathrm{Var}(GE) = \mathbb{E}[G^2E^2] = \mathbb{E}[G^2]\mathbb{E}[E^2] = \mathrm{Var}(G)\mathrm{Var}(E)$ under (A1)–(A2).

Write the Taylor expansion of $\varphi(Y) = \varphi(\alpha E + \beta G + \varepsilon)$ around ε:

$$\varphi(Y) = \varphi(\varepsilon) + \varphi'(\varepsilon)(\alpha E + \beta G) + \frac{\varphi''(\varepsilon)}{2}(\alpha E + \beta G)^2 + R_3, \tag{3}$$

where R_3 denotes remainder terms of order $(\alpha E + \beta G)^3$ and higher.

1.1 Deriving the OLS coefficients on the transformed scale

We now derive explicit formulas for each OLS coefficient by computing the relevant expectations term by term using the Taylor expansion (3).

Environment main effect $\hat{\alpha}^\varphi$

Computing $\mathbb{E}[E \cdot \varphi(Y)]$ term by term:

$$\mathbb{E}[E \cdot \varphi(\varepsilon)] = \mathbb{E}[E] \cdot \mathbb{E}[\varphi(\varepsilon)] = 0,$$

$$\mathbb{E}[E \cdot \varphi'(\varepsilon)(\alpha E + \beta G)] = \mathbb{E}[\varphi'(\varepsilon)] \cdot \mathbb{E}[E(\alpha E + \beta G)] = \mathbb{E}[\varphi'(\varepsilon)] \cdot \alpha \cdot \mathrm{Var}(E),$$

$$\begin{aligned}\mathbb{E}\left[E \cdot \frac{\varphi''(\varepsilon)}{2}(\alpha E + \beta G)^2\right] &= \frac{\mathbb{E}[\varphi''(\varepsilon)]}{2} \cdot \mathbb{E}[E(\alpha^2 E^2 + 2\alpha\beta GE + \beta^2 G^2)] \\ &= \frac{\mathbb{E}[\varphi''(\varepsilon)]}{2} \cdot \alpha^2 \mathbb{E}[E^3].\end{aligned}$$

Dividing by $\mathrm{Var}(E)$ per (1):

$$\hat{\alpha}^{\varphi} = \alpha \cdot \mathbb{E}[\varphi'(\varepsilon)] + \frac{\alpha^2 \mathbb{E}[E^3]}{2\mathrm{Var}(E)} \cdot \mathbb{E}[\varphi''(\varepsilon)] + O(\text{higher order}). \tag{4}$$

Genotype main effect $\hat{\beta}^{\varphi}$

By identical reasoning:

$$\hat{\beta}^{\varphi} = \beta \cdot \mathbb{E}[\varphi'(\varepsilon)] + \frac{\beta^2 \mathbb{E}[G^3]}{2\mathrm{Var}(G)} \cdot \mathbb{E}[\varphi''(\varepsilon)] + O(\text{higher order}). \tag{5}$$

Interaction coefficient $\hat{\gamma}^{\varphi}$

Computing $\mathbb{E}[GE \cdot \varphi(Y)]$ term by term:

$$\mathbb{E}[GE \cdot \varphi(\varepsilon)] = \mathbb{E}[GE] \cdot \mathbb{E}[\varphi(\varepsilon)] = 0.$$

$$\begin{aligned}\mathbb{E}[GE \cdot \varphi'(\varepsilon)(\alpha E + \beta G)] &= \mathbb{E}[\varphi'(\varepsilon)] \cdot \mathbb{E}[GE(\alpha E + \beta G)] \\ &= \mathbb{E}[\varphi'(\varepsilon)] \cdot (\alpha \mathbb{E}[GE^2] + \beta \mathbb{E}[G^2 E]) = 0.\end{aligned}$$

$$\begin{aligned}\mathbb{E}\left[GE \cdot \frac{\varphi''(\varepsilon)}{2}(\alpha E + \beta G)^2\right] &= \frac{\mathbb{E}[\varphi''(\varepsilon)]}{2} \cdot \mathbb{E}[GE(\alpha^2 E^2 + 2\alpha\beta GE + \beta^2 G^2)] \\ &= \frac{\mathbb{E}[\varphi''(\varepsilon)]}{2} \cdot (\alpha^2 \mathbb{E}[G]\mathbb{E}[E^3] + 2\alpha\beta \mathbb{E}[G^2]\mathbb{E}[E^2] + \beta^2 \mathbb{E}[G^3]\mathbb{E}[E]) \\ &= \frac{\mathbb{E}[\varphi''(\varepsilon)]}{2} \cdot 2\alpha\beta \cdot \mathrm{Var}(G) \cdot \mathrm{Var}(E) \\ &= \alpha\beta \cdot \mathbb{E}[\varphi''(\varepsilon)] \cdot \mathrm{Var}(G) \cdot \mathrm{Var}(E).\end{aligned}$$

Dividing by $\mathrm{Var}(G)\mathrm{Var}(E)$ per (2):

$$\hat{\gamma}^{\varphi} = \alpha\beta \cdot \mathbb{E}[\varphi''(\varepsilon)] + O(\text{higher order}). \tag{6}$$

Sign relationships

From (4) and (5), the leading-order terms give:

$$\mathrm{sgn}(\hat{\alpha}^{\varphi}) = \mathrm{sgn}(\varphi') \cdot \mathrm{sgn}(\alpha), \qquad \mathrm{sgn}(\hat{\beta}^{\varphi}) = \mathrm{sgn}(\varphi') \cdot \mathrm{sgn}(\beta).$$

Therefore:

$$\operatorname{sgn}(\hat{\alpha}^{\varphi}) \cdot \operatorname{sgn}(\hat{\beta}^{\varphi}) = \operatorname{sgn}(\varphi')^2 \cdot \operatorname{sgn}(\alpha) \cdot \operatorname{sgn}(\beta) = \operatorname{sgn}(\alpha\beta), \tag{7}$$

since $\operatorname{sgn}(\varphi')^2 = 1$. This shows that the sign of φ' (whether the transformation is increasing or decreasing) cancels when we take the product of the observed main effects.

1.2 Proof of Theorem 1 (Sign-consistent interaction property)

The following corresponds to Theorem 1 in the main manuscript.

Theorem 1 (Sign-consistent interaction property)**.** *Under assumptions (A1)–(A6), if* $Y = \alpha E + \beta G + \varepsilon$ *has no interaction on the original scale, then regressing* $\varphi(Y)$ *on* (G, E, GE) *yields:*

$$\hat{\gamma}^{\varphi} = \alpha\beta \cdot \mathbb{E}[\varphi''(\varepsilon)] + O(\textit{higher order}). \tag{8}$$

Consequently, the sign rule from the main manuscript equation (1) holds:

$$\operatorname{sgn}(\hat{\gamma}^{\varphi}) = \operatorname{sgn}(\varphi'') \cdot \operatorname{sgn}(\hat{\alpha}^{\varphi}) \cdot \operatorname{sgn}(\hat{\beta}^{\varphi}).$$

Note that the induced interaction coefficient $\hat{\gamma}^{\varphi}$ depends only on the product $\alpha\beta$ and the expected curvature $\mathbb{E}[\varphi''(\varepsilon)]$, and not on whether φ is increasing or decreasing (the sign of φ'), nor on skewness of G or E. The distinction between increasing and decreasing transformations is absorbed into the observed coefficients $\hat{\alpha}^{\varphi}$ and $\hat{\beta}^{\varphi}$.

Proof. The interaction coefficient formula (8) follows from (6). For the sign rule, combining (6) with (7):

$$\operatorname{sgn}(\hat{\gamma}^{\varphi}) = \operatorname{sgn}(\alpha\beta) \cdot \operatorname{sgn}(\mathbb{E}[\varphi''(\varepsilon)]) = \operatorname{sgn}(\hat{\alpha}^{\varphi}) \cdot \operatorname{sgn}(\hat{\beta}^{\varphi}) \cdot \operatorname{sgn}(\varphi''). \qquad \square$$

1.3 The nonlinearity coefficient κ

For symmetric predictors, we can express the interaction in a particularly elegant form. When $\mathbb{E}[E^3] = 0$, equation (4) simplifies to:

$$\hat{\alpha}^{\varphi} \approx \alpha \cdot \mathbb{E}[\varphi'(\varepsilon)].$$

Similarly, when $\mathbb{E}[G^3] = 0$, equation (5) simplifies to $\hat{\beta}^{\varphi} \approx \beta \cdot \mathbb{E}[\varphi'(\varepsilon)]$.

We now introduce the scale-free nonlinearity coefficient κ:

Corollary 1 (Nonlinearity coefficient for symmetric predictors)**.** *Under assumptions (A1)–(A6), if* $\mathbb{E}[G^3] = \mathbb{E}[E^3] = 0$ *(symmetric predictors), then the interaction from Theorem 1 can be expressed as:*

$$\hat{\gamma}^{\varphi} \approx \hat{\alpha}^{\varphi} \cdot \hat{\beta}^{\varphi} \cdot \kappa$$

where the ***nonlinearity coefficient*** *is:*

$$\kappa := \frac{\mathbb{E}[\varphi''(\varepsilon)]}{(\mathbb{E}[\varphi'(\varepsilon)])^2}.$$

Proof. Under the symmetric predictor assumption:

$$\hat{\alpha}^\varphi \approx \alpha \cdot \mathbb{E}[\varphi'(\varepsilon)], \quad \hat{\beta}^\varphi \approx \beta \cdot \mathbb{E}[\varphi'(\varepsilon)], \quad \hat{\gamma}^\varphi \approx \alpha\beta \cdot \mathbb{E}[\varphi''(\varepsilon)].$$

Therefore:

$$\hat{\alpha}^\varphi \cdot \hat{\beta}^\varphi \cdot \kappa = \alpha\beta \cdot (\mathbb{E}[\varphi'(\varepsilon)])^2 \cdot \frac{\mathbb{E}[\varphi''(\varepsilon)]}{(\mathbb{E}[\varphi'(\varepsilon)])^2} = \alpha\beta \cdot \mathbb{E}[\varphi''(\varepsilon)] = \hat{\gamma}^\varphi.$$

□

This yields the sign consistency property:

$$\text{sgn}(\hat{\gamma}^\varphi) = \text{sgn}(\kappa) \cdot \text{sgn}(\hat{\alpha}^\varphi) \cdot \text{sgn}(\hat{\beta}^\varphi) = \text{sgn}(\varphi'') \cdot \text{sgn}(\hat{\alpha}^\varphi) \cdot \text{sgn}(\hat{\beta}^\varphi).$$

1.4 Proof of Corollary 1 (Sign consistency for diploid genotypes)

The following corresponds to Corollary 1 in the main manuscript.

Corollary 2 (Sign consistency for diploid genotypes)**.** *The sign rule extends to diploid genotypes $G \in \{0, 1, 2\}$ under Hardy-Weinberg equilibrium, with E any random variable satisfying the independence and moment conditions (A1)–(A3).*

Theorem 1 applies directly to the case where G follows Hardy-Weinberg equilibrium and E is binary or continuous. The interaction coefficient formula $\hat{\gamma}^\varphi \approx \alpha\beta \cdot \mathbb{E}[\varphi''(\varepsilon)]$ holds regardless of the specific distributions of G and E; only the main effect formulas require distribution-dependent corrections.

Let $G \in \{0, 1, 2\}$ follow Hardy-Weinberg equilibrium with minor allele frequency p:

$$\Pr(G = 0) = (1 - p)^2, \quad \Pr(G = 1) = 2p(1 - p), \quad \Pr(G = 2) = p^2.$$

The first four moments are computed

$$\begin{aligned}
\mathbb{E}[G] &= 2p, \\
\text{Var}(G) &= 2p(1 - p), \\
\mathbb{E}[(G - \mathbb{E}[G])^3] &= 2p(1 - p)(1 - 2p), \\
\mathbb{E}[(G - \mathbb{E}[G])^4] &= 2p(1 - p)(1 + (1 - 2p)^2).
\end{aligned}$$

After centering $G \leftarrow G - 2p$:

$$\mathbb{E}[G^3] = 2p(1 - p)(1 - 2p).$$

Note that the third moment $\mathbb{E}[G^3] = 0$ only when $p = 1/2$.

For typical allele frequencies ($p \neq 1/2$), a skewness correction is needed for the genotype main effect:

$$\hat{\beta}^{\varphi} \approx \beta \cdot \mathbb{E}[\varphi'(\varepsilon)] + \frac{\beta^2(1-2p)}{2} \cdot \mathbb{E}[\varphi''(\varepsilon)].$$

However, since the correction is $O(\beta^2)$ while the leading term is $O(\beta)$, for small effect sizes the approximation $\hat{\beta}^{\varphi} \approx \beta \cdot \mathbb{E}[\varphi'(\varepsilon)]$ remains accurate.

The interaction coefficient:

$$\hat{\gamma}^{\varphi} \approx \alpha\beta \cdot \mathbb{E}[\varphi''(\varepsilon)]$$

remains exact to second order regardless of p.

2 Transformations with dependent G and E

We now extend the analysis to allow G and E to be correlated. This section provides the proof of Theorem 2 and Corollary 2 from the main manuscript.

We replace assumption (A2) with:

(A2') **Possible dependence:** $\mathrm{Cov}(G, E) = \rho\sigma_G\sigma_E$ where $\rho \in [-1, 1]$

When $\rho = 0$, this reduces to Part I. All other assumptions (A1), (A3)–(A6) remain.

With dependence, the predictors G, E, and GE are no longer orthogonal: $\mathbb{E}[GE] = \rho\sigma_G\sigma_E \neq 0$, and cross-moments like $\mathbb{E}[G^2E]$ and $\mathbb{E}[GE^2]$ no longer vanish. Consequently, the OLS coefficients no longer decompose as simple univariate projections—they couple through the normal equations, and the interaction coefficient receives contributions from both the correlation structure and the transformation curvature.

2.1 Proof of Theorem 2 (Sign consistency for correlated G and E)

The following corresponds to Theorem 2 in the main manuscript.

Theorem 2 (Sign consistency for correlated G and E)**.** *Under assumptions (A1), (A2'), (A3)–(A6), if $Y = \alpha E + \beta G + \varepsilon$ on the original scale (no true interaction), then regressing $\varphi(Y)$ on $(1, G, E, GE)$ yields an interaction coefficient:*

$$\hat{\gamma}^{\varphi} \approx \underbrace{\gamma_{corr}}_{\text{correlation effect}} + \underbrace{\alpha\beta \cdot \mathbb{E}[\varphi''(\varepsilon)]}_{\text{transformation effect}}.$$

where γ_{corr} arises from the non-orthogonality of predictors. The sign rule holds when the transformation effect dominates: $|\alpha\beta \cdot \mathbb{E}[\varphi'']| > |\gamma_{corr}|$.

When $\rho = 0$ (i.e., $G \perp\!\!\!\perp E$), we have $\gamma_{corr} = 0$ and this reduces to Theorem 1.

Proof. The interaction coefficient receives contributions from two sources.

Correlation effect. Even without transformation ($\varphi = \text{id}$), the non-orthogonality of predictors means the OLS projection onto GE picks up contributions from the correlated structure. Terms involving $\mathbb{E}[GE]$, $\mathbb{E}[G^2E]$, and $\mathbb{E}[GE^2]$ contribute to γ_{corr} through the coupled normal equations.

Transformation effect. The curvature φ'' generates an interaction term exactly as in Section 1. The second-order Taylor expansion contributes $\alpha\beta \cdot \mathbb{E}[\varphi''(\varepsilon)]$ to the interaction coefficient.

When $\rho = 0$, we have $\mathbb{E}[GE] = \mathbb{E}[G^2E] = \mathbb{E}[GE^2] = 0$, the predictors become orthogonal, $\gamma_{\text{corr}} = 0$, and we recover Theorem 1. □

Corollary 3 (Dominance of transformation effect)**.** *This corresponds to Corollary 2 in the main manuscript.*

For small gene-environment correlations or strong curvature $|\varphi''|$, the sign rule approximately holds:

$$\text{sgn}(\hat{\gamma}^{\varphi}) \approx \text{sgn}(\varphi'') \cdot \text{sgn}(\hat{\alpha}^{\varphi}) \cdot \text{sgn}(\hat{\beta}^{\varphi}).$$

In practice, if observed interactions are strongly sign-consistent across many variants, the transformation effect likely dominates any correlation-induced deviations.

Proof. From Theorem 2:

$$\hat{\gamma}^{\varphi} = \gamma_{\text{corr}} + \alpha\beta \cdot \mathbb{E}[\varphi''(\varepsilon)] + O(\text{higher order}).$$

When $|\gamma_{\text{corr}}| \ll |\alpha\beta \cdot \mathbb{E}[\varphi''(\varepsilon)]|$, the transformation effect dominates:

$$\text{sgn}(\hat{\gamma}^{\varphi}) \approx \text{sgn}(\alpha\beta \cdot \mathbb{E}[\varphi''(\varepsilon)]) = \text{sgn}(\alpha) \cdot \text{sgn}(\beta) \cdot \text{sgn}(\varphi'').$$

Conversely, when $|\gamma_{\text{corr}}| \gg |\alpha\beta \cdot \mathbb{E}[\varphi''(\varepsilon)]|$, we have $\text{sgn}(\hat{\gamma}^{\varphi}) \approx \text{sgn}(\gamma_{\text{corr}})$, which depends on the sign and magnitude of the gene-environment correlation and the associated moment structure. □

2.1.1 Implications

Theorem 2 and Corollary 3 clarify how gene-environment correlation affects the sign rule. When $\rho = 0$, we recover Theorem 1 exactly and the sign rule holds without qualification. When $\rho \neq 0$, there is a "baseline" interaction from correlation that exists even without transformation. The term γ_{corr} arises from the non-orthogonality of the design matrix and captures how the correlation between G and E manifests as an interaction coefficient.

The sign of the observed interaction,

$$\text{sgn}(\hat{\gamma}^{\varphi}) = \text{sgn}\big(\gamma_{\text{corr}} + \alpha\beta \cdot \mathbb{E}[\varphi''(\varepsilon)]\big),$$

depends on the relative magnitudes of the two contributions. Three regimes emerge. In the *transformation-dominated* regime where $|\alpha\beta \cdot \mathbb{E}[\varphi''(\varepsilon)]| \gg |\gamma_{\text{corr}}|$, the sign rule from Section 1 applies. In the *correlation-dominated* regime where $|\gamma_{\text{corr}}| \gg |\alpha\beta \cdot \mathbb{E}[\varphi''(\varepsilon)]|$, the sign is determined

by γ_{corr}. In the *intermediate* regime where the two terms are comparable, the sign depends on whether they reinforce or cancel.

3 Endogenous Treatment Effects

This section proves Theorem 3 (opposite-sign rule for endogenous treatment) and derives a quantity analogous to the nonlinearity coefficient κ for transformations.

Consider an environmental exposure E assigned based on a threshold rule:

$$E = \begin{cases} 1 & \text{if } Y > t, \\ 0 & \text{otherwise,} \end{cases}$$

where $Y \sim \mathcal{N}(\mu, 1)$ is the pre-treatment phenotype and t is the treatment threshold. The observed phenotype is $\tilde{Y} = Y + \alpha E$, where α is the treatment effect.

For a genetic variant G_j with effect β_j on Y, we have conditional means $\mu_j^0 = \mathbb{E}[Y|G_j = 0]$ and $\mu_j^1 = \mathbb{E}[Y|G_j = 1]$, with $\beta_j = \mu_j^1 - \mu_j^0$.

Define $P_0 := t - \mu_j^0$ and $P_1 := t - \mu_j^1$, so $P_1 - P_0 = -\beta_j$.

From the main text derivation, the estimated effects in the regression $\tilde{Y} = \hat{\mu}_j + \hat{\alpha}_j E + \hat{\beta}_j G_j + \hat{\gamma}_j G_j E + \varepsilon_j$ are:

$$\begin{aligned} \hat{\beta}_j &= \lambda(P_0) - \lambda(P_1) + (P_0 - P_1), \\ \hat{\gamma}_j &= \lambda(-P_1) - \lambda(-P_0) - (\lambda(P_0) - \lambda(P_1)), \end{aligned}$$

where $\lambda(x) = \phi(x)/\Phi(x)$ is the inverse Mill's ratio.

For small genetic effects ($|\beta_j| \ll 1$), expand around P_0. Let $\delta := \beta_j = \mu_j^1 - \mu_j^0$, so $P_1 = P_0 - \delta$.

The Taylor expansion of the genetic effect is:

$$\begin{aligned} \hat{\beta}_j &= \lambda(P_0) - \lambda(P_0 - \delta) + \delta \\ &\approx \lambda'(P_0) \cdot \delta + \delta \\ &= \delta \cdot (1 + \lambda'(P_0)). \end{aligned}$$

Next, the Taylor expansion of $\hat{\gamma}_j$ is computed

$$\begin{aligned} \hat{\gamma}_j &= \lambda(-P_0 + \delta) - \lambda(-P_0) - (\lambda(P_0) - \lambda(P_0 - \delta)) \\ &\approx \lambda'(-P_0) \cdot \delta - \lambda'(P_0) \cdot \delta \\ &= \delta \cdot (\lambda'(-P_0) - \lambda'(P_0)). \end{aligned}$$

We define the **endogenous treatment coefficient**:

$$\eta(P) := \frac{\lambda'(-P) - \lambda'(P)}{1 + \lambda'(P)}$$

Taking the ratio of the above Taylor expansions:

$$\hat{\gamma}_j \approx \eta(P_0) \cdot \hat{\beta}_j \tag{9}$$

The inverse Mill's ratio λ has derivative $\lambda'(x) = -\lambda(x)(x + \lambda(x))$.

Theorem 3 (η is always negative for $P > 0$)**.** *For $P > 0$, we have $\eta(P) < 0$.*

Proof. Since λ is strictly convex ($\lambda'' > 0$), we have λ' strictly increasing. For $P > 0$: $-P < P$, so $\lambda'(-P) < \lambda'(P) < 0$ and therefore $\lambda'(-P) - \lambda'(P) < 0$. Since $\lambda'(P) < 0$ and $|\lambda'(P)| < 1$ for reasonable P, we have $1 + \lambda'(P) > 0$. Thus $\eta(P) < 0$. □

The following corresponds to Theorem 3 in the main manuscript.

Theorem 4 (Opposite-sign rule for endogenous treatment)**.** *When $P_0 > 0$ (treatment threshold above reference mean):*

$$\text{sgn}(\hat{\gamma}_j) = -\text{sgn}(\hat{\beta}_j).$$

Proof. From (9) and the fact that $\eta(P) < 0$ for $P > 0$:

$$\text{sgn}(\hat{\gamma}_j) = \text{sgn}(\eta) \cdot \text{sgn}(\hat{\beta}_j) = (-1) \cdot \text{sgn}(\hat{\beta}_j) = -\text{sgn}(\hat{\beta}_j).$$

□
