# Supplementary material for: The geometry of G × E: How scaling and endogenous treatment effects shape interaction direction
Source: PLoS Genet. 2026 Apr 1;22(4):e1012073. doi: 10.1371/journal.pgen.1012073 (PMC13043064; doi:10.1371/journal.pgen.1012073)
Supplement: S1 File — Supplementary notes. (PDF) [file pgen.1012073.s002.pdf]

Supporting Information

The geometry of $G \times E$: how scaling and endogenous treatment effects shape interaction direction

Michal Sadowski, Andy W. Dahl, Noah Zaitlen, and Richard Border

1 The logistic transformation

Consider the logistic function:

$$\varphi(x) = \frac{1}{1 + e^{-(x - x_0)}},$$

and four points $P_{0,0}$, $P_{0,1}$, $P_{1,0}$, and $P_{1,1}$, such that $P_{0,1} - P_{0,0} = P_{1,1} - P_{1,0} = d$ (Fig S3A).

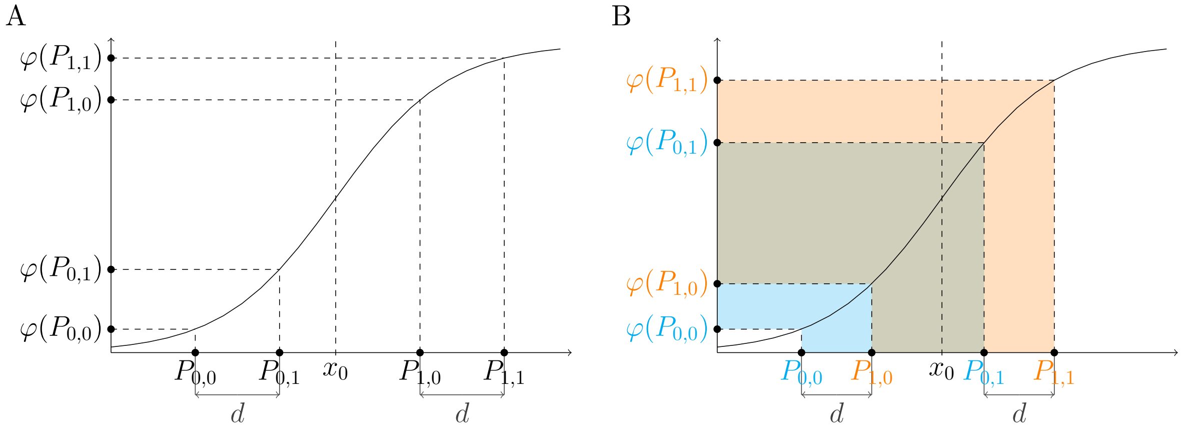

Fig S3. The effect of the values $P_{0,0}$, $P_{0,1}$, $P_{1,0}$, and $P_{1,1}$ on the sign of expression: $\varphi(P_{1,1}) - \varphi(P_{1,0}) - (\varphi(P_{0,1}) - \varphi(P_{0,0}))$, where φ is the logistic function. (A) When the order of the input values is $P_{0,0} < P_{0,1} < x_0 < P_{1,0} < P_{1,1}$. (B) When the order of the input values is $P_{0,0} < P_{1,0} < x_0 < P_{0,1} < P_{1,1}$.

We examine the sign of $\hat{\gamma}^{\varphi}$, the value defined below, as a function of the values of points $P_{0,0}$, $P_{0,1}$, $P_{1,0}$, and $P_{1,1}$:

$$\operatorname{sgn}\hat{\gamma}^{\varphi} = \operatorname{sgn}(\varphi(P_{1,1}) - \varphi(P_{1,0}) - (\varphi(P_{0,1}) - \varphi(P_{0,0}))).$$

There are six possible cases (without loss of generality, we assume that $P_{0,1} - P_{0,0} > 0$ and $P_{1,0} - P_{0,0} > 0$):

1. If all the points $P_{a,b}$ are smaller than x_0, transforming them with φ is equivalent to transforming them with an increasing convex down function. As shown in Methods, this results in $\hat{\gamma}^{\varphi} > 0$ (Table 2 and Fig 2A).

2. If all the points $P_{a,b}$ are larger than x_0, transforming them with φ is equivalent to transforming them with an increasing convex up function. As shown in Methods, this results in $\hat{\gamma}^{\varphi} < 0$ (Table 2 and Fig 2C).

3. If $P_{0,0}$ and $P_{0,1}$ are smaller than x_0, but $P_{1,0}$ and $P_{1,1}$ are larger than x_0, the sign of $\hat{\gamma}^{\varphi}$ depends on the relation between distances $|P_{0,1} - x_0|$ and $|P_{1,0} - x_0|$ (Fig 4A). Note that $\varphi'(x_0 + x) = \varphi'(x_0 - x)$, and that the derivative of φ decreases as the distance from x_0 increases. Recall also our assumption that $P_{0,1} - P_{0,0} = P_{1,1} - P_{1,0}$. Therefore:

   - If $|P_{0,1} - x_0| > |P_{1,0} - x_0|$, then $\int_{P_{0,0}}^{P_{0,1}} \varphi'(x)dx < \int_{P_{1,0}}^{P_{1,1}} \varphi'(x)dx$. Consequently, $\hat{\gamma}^{\varphi} > 0$.
   - If $|P_{0,1} - x_0| < |P_{1,0} - x_0|$, then $\int_{P_{0,0}}^{P_{0,1}} \varphi'(x)dx > \int_{P_{1,0}}^{P_{1,1}} \varphi'(x)dx$. Consequently, $\hat{\gamma}^{\varphi} < 0$.

4. Similarly as in point 3, if $P_{0,0}$ and $P_{1,0}$ are smaller than x_0, but $P_{0,1}$ and $P_{1,1}$ are larger than x_0, the sign of $\hat{\gamma}^{\varphi}$ depends on the relation between distances $|P_{0,1} - x_0|$ and $|P_{1,0} - x_0|$ (Fig S3B). Note that:

   $$\text{sgn}\hat{\gamma}^{\varphi} = \text{sgn}(\varphi(P_{1,1}) - \varphi(P_{1,0}) - (\varphi(P_{0,1}) - \varphi(P_{0,0}))) = \text{sgn}(\varphi(P_{1,1}) - \varphi(P_{0,1}) - (\varphi(P_{1,0}) - \varphi(P_{0,0}))).$$

   We use the same argument as in point 3, but this time the differences we compare are $P_{1,1} - P_{0,1}$ and $P_{1,0} - P_{0,0}$, instead of $P_{1,1} - P_{1,0}$ and $P_{0,1} - P_{0,0}$. This comparison yields:

   - If $|P_{0,1} - x_0| > |P_{1,0} - x_0|$, then $\hat{\gamma}^{\varphi} < 0$.
   - If $|P_{0,1} - x_0| < |P_{1,0} - x_0|$, then $\hat{\gamma}^{\varphi} > 0$.

5. If $P_{0,0}$ is smaller than x_0, and $P_{0,1}$, $P_{1,0}$ and $P_{1,1}$ are larger than x_0, then—since φ' decreases as the distance from x_0 increases—$\varphi(P_{0,1}) - \varphi(P_{0,0})$ is greater than $\varphi(P_{1,1}) - \varphi(P_{1,0})$, and consequently $\hat{\gamma}^{\varphi} < 0$.

6. Conversely to point 5, when only $P_{1,1}$ is larger than x_0, and $P_{0,0}$, $P_{0,1}$ and $P_{1,0}$ are smaller than x_0, $\varphi(P_{0,1}) - \varphi(P_{0,0})$ is smaller than $\varphi(P_{1,1}) - \varphi(P_{1,0})$, and consequently $\hat{\gamma}^{\varphi} > 0$.

2 Heteroskedasticity

Heterogeneity of phenotype variance across values of genetic and/or environmental factors (heteroskedasticity) can produce false $G \times E$ [1]. Since large differences of phenotypic variance between environments are common, conditional heteroskedasticity is an important source of statistical artifacts in $G \times E$ studies [2, 3]. It is therefore important that interaction models account for it.

In the presence of environment-conditional heteroskedasticity, testing multiple genetic variants for interaction with the environmental factor in a simple linear regression model results in an inflated or deflated false positive rate (FPR), depending on the relation between group size and phenotypic variation [2]. We demonstrate this in simulation and derive a formula revealing this relation in a univariate model.

Consider a binary environmental variable that divides observations into two groups of sizes n_0 and n_1, and phenotype variances σ_0^2 and σ_1^2. We simulated data for 10,000 such observations. The phenotype was drawn from Gaussian distributions with mean zero and corresponding group variances. We independently drew 200 genotypes from a binomial distribution $B(2, p)$ with p representing the minor allele frequency, which was drawn uniformly from a range between 0.1 and 0.5. For each genotype, two $G \times E$ models were fitted: 1) a simple linear regression model that included the genotype, the environmental factor and the product of those two as covariates (OLS), and 2) a double generalized model (DGLM) that used the same covariates to model the mean effects, and the environmental variable to model the variance effects.

We ran this simulation for selected values of the ratio σ_1^2/σ_0^2, and calculated the FPR for the $G \times E$ effect as the proportion of simulations where the nominal p-value for this effect was less than 0.05 (Fig S4A). The simulation shows that if the smaller (larger) group is characterized by the larger (smaller) variance of the response, the FPR for the OLS is inflated (deflated). Within a realistic range of parameter values, the FPR can reach zero or increase threefold. On the other hand, if the groups have equal sizes, the model is well calibrated. Fig S4B shows p-value distributions for one such simulation with realistic variance ratios $\sigma_1^2/\sigma_0^2 = 3$ and $\sigma_1^2/\sigma_0^2 = 1/3$ [3].

Below is an analytical demonstration of how this bias arises. Consider the following linear regression model:

$$\underbrace{\begin{bmatrix} \boldsymbol{y}_0 \\ \boldsymbol{y}_1 \end{bmatrix}}_{\boldsymbol{y}} = \hat{\beta} \underbrace{\begin{bmatrix} \boldsymbol{x}_0 \\ \boldsymbol{x}_1 \end{bmatrix}}_{\boldsymbol{x}} + \boldsymbol{\epsilon}, \tag{1}$$

where:

- $\boldsymbol{y}$ is a size N vector generated by concatenating vectors $\boldsymbol{y}_0$ and $\boldsymbol{y}_1$, and centering. Individually, $\boldsymbol{y}_0$ ($\boldsymbol{y}_1$) is a vector of size n_0 (n_1), whose elements were independently drawn from a Gaussian distribution with mean zero and variance σ_0^2 (σ_1^2).
- $\boldsymbol{x}$ is a size N vector generated by concatenating a size n_0 vector of zeros ($\boldsymbol{x}_0$) and a size n_1 vector of ones ($\boldsymbol{x}_1$), and centering. We use $k_0 := -n_1/N$ and $k_1 := 1 - n_1/N = n_0/N$ to refer to the values of $\boldsymbol{x}_0$ and $\boldsymbol{x}_1$, respectively, after centering.
- $\boldsymbol{\epsilon}$ is a size N vector of residuals.

The variance of the estimate $\hat{\beta}$ is given as:

$$\text{Var}[\hat{\beta}] = (\boldsymbol{x}^T\boldsymbol{x})^{-2}\boldsymbol{x}^T\text{Var}[\boldsymbol{y}]\boldsymbol{x}. \tag{2}$$

A B

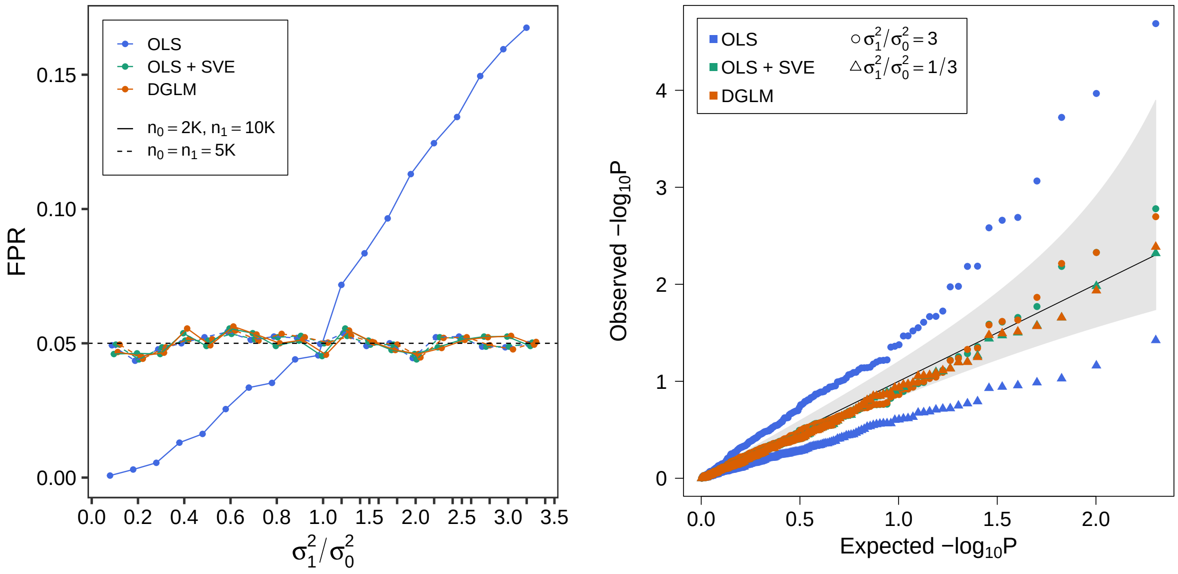

Fig S4. Evaluation of the type I error rate for the $G \times E$ effect estimated with the OLS model, the OLS model using robust standard errors (OLS + SVE) and the DGLM. (A) False positive rate (FPR) of $G \times E$ as a function of the ratio between phenotype variances in two environments: unexposed (of size n_0 and phenotype variance σ_0^2), and exposed (of size n_1 and phenotype variance σ_1^2). The nominal FPR of 5% is marked by the black dashed line. (B) Quantile-quantile plot comparing the null expected p-values (x-axis) to the observed $G \times E$ p-values when $\sigma_1^2/\sigma_0^2 = 3$ (circles) and when $\sigma_1^2/\sigma_0^2 = 1/3$ (triangles).

Given the assumptions of model (1), it can be expressed as:

$$\mathrm{Var}[\hat{\beta}] = \frac{n_0 k_0^2 \sigma_0^2 + n_1 k_1^2 \sigma_1^2}{(n_0 k_0^2 + n_1 k_1^2)^2} =: v_{\text{true}}.$$

Recall that $k_0 = -n_1/N$, and $k_1 = n_0/N$. We can make those substitutions to simplify the above expression to:

$$v_{\text{true}} = \frac{n_0 \frac{n_1^2}{N^2} \sigma_0^2 + n_1 \frac{n_0^2}{N^2} \sigma_1^2}{\left(n_0 \frac{n_1^2}{N^2} + n_1 \frac{n_0^2}{N^2} \right)^2} = \frac{\frac{n_0 n_1}{N^2}(n_1 \sigma_0^2 + n_0 \sigma_1^2)}{\frac{n_0^2 n_1^2}{N^2}} = \frac{\sigma_0^2}{n_0} + \frac{\sigma_1^2}{n_1}. \tag{3}$$

However, the simple linear regression (SLR) model assumes homoskedasticity ($\mathrm{Var}[\boldsymbol{y}] = \sigma^2 \boldsymbol{I}$, where $\boldsymbol{I}$ is an identity matrix), in which case (2) simplifies to:

$$v_{\text{SLR}} = \frac{\sigma^2}{n_0 k_0^2 + n_1 k_1^2}. \tag{4}$$

The parameter σ^2 is then estimated as:

$$\begin{aligned}\hat{\sigma}^2 &= \frac{(\boldsymbol{y} - \boldsymbol{x}\hat{\beta})^T(\boldsymbol{y} - \boldsymbol{x}\hat{\beta})}{N} = \frac{\boldsymbol{y}^T\boldsymbol{y} - 2\hat{\beta}^T\boldsymbol{x}^T\boldsymbol{y} + \hat{\beta}^T\boldsymbol{x}^T\boldsymbol{x}\hat{\beta}}{N} = \frac{\boldsymbol{y}^T\boldsymbol{y} - \boldsymbol{y}^T\boldsymbol{x}(\boldsymbol{x}^T\boldsymbol{x})^{-1}\boldsymbol{x}^T\boldsymbol{y}}{N} \\ &= \frac{\boldsymbol{y}^T\boldsymbol{y} - (\boldsymbol{x}^T\boldsymbol{x})^{-1}(\boldsymbol{x}^T\boldsymbol{y})^2}{N},\end{aligned}$$

where we use the fact that $\hat{\beta} = (\boldsymbol{x}^T\boldsymbol{x})^{-1}\boldsymbol{x}^T\boldsymbol{y}$. Given the assumptions of model (1), this can be computed as:

$$\begin{aligned}\hat{\sigma}^2 =& \frac{\sum_{i=1}^{n_0} y_i^2 + \sum_{i=n_0+1}^{N} y_i^2}{N} - \frac{(k_0 \sum_{i=1}^{n_0} y_i + k_1 \sum_{i=n_0+1}^{N} y_i)^2}{N(n_0 k_0^2 + n_1 k_1^2)} \\ =& \frac{n_0 \mathbb{E}[\boldsymbol{y}_0^2] + n_1 \mathbb{E}[\boldsymbol{y}_1^2]}{N} - \frac{k_0^2 n_0^2 \mathbb{E}[\boldsymbol{y}_0]^2 + 2k_0 k_1 n_0 n_1 \mathbb{E}[\boldsymbol{y}_0]\mathbb{E}[\boldsymbol{y}_1] + k_1^2 n_1^2 \mathbb{E}[\boldsymbol{y}_1]^2}{N(n_0 k_0^2 + n_1 k_1^2)}\end{aligned}$$

where $\hat{\sigma}_0^2 \coloneqq \mathbb{E}[\boldsymbol{y}_0^2] - \mathbb{E}[\boldsymbol{y}_0]^2$, $\hat{\sigma}_1^2 \coloneqq \mathbb{E}[\boldsymbol{y}_1^2] - \mathbb{E}[\boldsymbol{y}_1]^2$, $\hat{\mu}_0 \coloneqq \mathbb{E}[\boldsymbol{y}_0]$, and $\hat{\mu}_1 \coloneqq \mathbb{E}[\boldsymbol{y}_1]$. This expression is simplified when substituting k_0 for $-n_1/N$, and k_1 for n_0/N:

$$\begin{aligned}\hat{\sigma}^2 &= \frac{n_0 n_1 \hat{\sigma}_0^2 + n_0 n_1 \hat{\sigma}_1^2 + n_0^2 \mathbb{E}[\boldsymbol{y}_0^2] + n_1^2 \mathbb{E}[\boldsymbol{y}_1^2] + 2n_0 n_1 \hat{\mu}_0 \hat{\mu}_1}{N^2} = \frac{N n_0 \hat{\sigma}_0^2 + N n_1 \hat{\sigma}_1^2 + n_0^2 \hat{\mu}_0^2 + n_1^2 \hat{\mu}_1^2 + 2n_0 n_1 \hat{\mu}_0 \hat{\mu}_1}{N^2} \\ &= \frac{n_0 \hat{\sigma}_0^2 + n_1 \hat{\sigma}_1^2}{N} + \left(\frac{n_0 \hat{\mu}_0 + n_1 \hat{\mu}_1}{N} \right)^2 = \frac{n_0 \hat{\sigma}_0^2 + n_1 \hat{\sigma}_1^2}{N},\end{aligned}$$

where the last equality follows from the fact that $\boldsymbol{y}$ is centered. Therefore, following (4), the SLR estimates the variance of $\hat{\beta}$ as:

$$\hat{v}_{\text{SLR}} = \frac{n_0 \hat{\sigma}_0^2 + n_1 \hat{\sigma}_1^2}{N} \frac{N}{n_0 n_1} = \frac{\hat{\sigma}_0^2}{n_1} + \frac{\hat{\sigma}_1^2}{n_0},$$

which is different from the estimate of the true variance, $\hat{v}_{\text{true}} = \hat{\sigma}_0^2/n_0 + \hat{\sigma}_1^2/n_1$, as derived in (3). In particular, examination of the difference between these estimates:

$$d_v := \hat{v}_{\text{true}} - \hat{v}_{\text{SLR}} = \frac{n_1\hat{\sigma}_0^2 + n_0\hat{\sigma}_1^2}{n_0 n_1} - \frac{n_0\hat{\sigma}_0^2 + n_1\hat{\sigma}_1^2}{n_0 n_1} = \frac{n_1 - n_0}{n_0 n_1}(\hat{\sigma}_0^2 - \hat{\sigma}_1^2),$$

shows that:

- if $n_0 > n_1$ and $\sigma_0^2 < \sigma_1^2$, then $d_v > 0$, which means that the t-statistic computed with SLR is inflated;
- if $n_0 > n_1$ and $\sigma_0^2 > \sigma_1^2$, then $d_v < 0$, which means that the t-statistic computed with SLR is deflated;
- if $n_0 = n_1$, then $\hat{v}_{\text{true}} = \hat{v}_{\text{SLR}}$, which means that the t-statistic computed with SLR is well-calibrated.

The described bias can be removed by incorporating robust standard errors estimated with the sandwich variance estimator (SVE) into the OLS (Fig S4). Alternatively, the data can be correctly modeled with the DGLM.

References

1. Paré, G., Cook, N. R., Ridker, P. M. & Chasman, D. I. On the use of variance per genotype as a tool to identify quantitative trait interaction effects: A report from the women's genome health study. *PLoS Genetics* **6,** 1–10 (2010).
2. Almli, L. M. *et al.* Correcting systematic inflation in genetic association tests that consider interaction effects application to a genome-wide association study of posttraumatic stress disorder. *JAMA Psychiatry* **71,** 1392–1399 (2014).
3. Sadowski, M. *et al.* Characterizing the genetic architecture of drug response using gene-context interaction methods. *Cell Genomics* **4,** 100722 (2024).
