# Supplementary figures and images for: The geometry of G × E: How scaling and endogenous treatment effects shape interaction direction
Source: PLoS Genet. 2026 Apr 1;22(4):e1012073. doi: 10.1371/journal.pgen.1012073 (PMC13043064; doi:10.1371/journal.pgen.1012073)

A

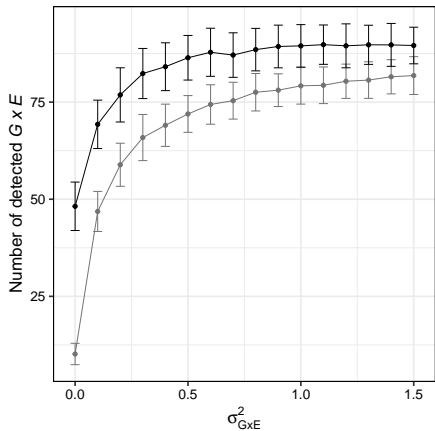

B

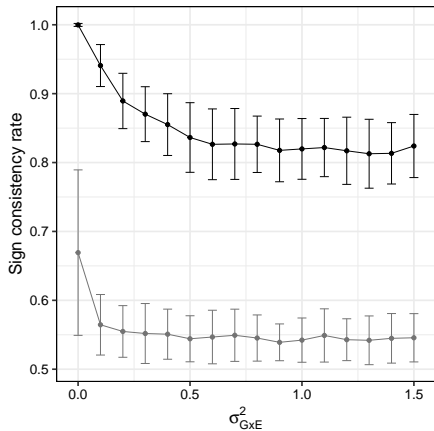

Supplement: S1 Fig — A: Number of detected G × E effects for outcomes on the original and transformed scales as a function of the variance of the simulated G × E effects, σGxE2. B: Estimated rate of sign consistency for outcomes on the original and transformed scales as a function of the variance of the simulated G × E effects, σGxE2. The sign consistency rate was defined as the proportion of G × E effects exhibiting the more prevalent sign relationship with their corresponding main effects. Due to this definition, sign consistency rate for the untransformed outcome may exceed 0.5. (PDF) [file pgen.1012073.s003.pdf]

A

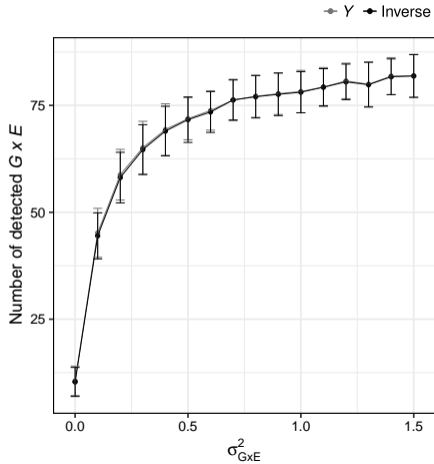

B

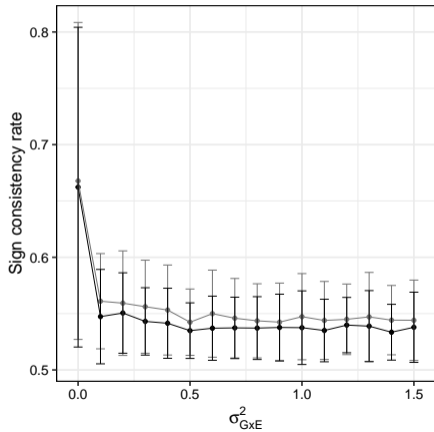

Supplement: S2 Fig — A: Number of detected G × E effects for outcomes on the original and transformed scales as a function of the variance of the simulated G × E effects, σGxE2. B: Estimated rate of sign consistency for outcomes on the original and transformed scales as a function of the variance of the simulated G × E effects, σGxE2. The sign consistency rate was defined as the proportion of G × E effects exhibiting the more prevalent sign relationship with their corresponding main effects. Due to this definition, sign consistency rate for the untransformed outcome may exceed 0.5. (PDF) [file pgen.1012073.s004.pdf]
